# Interaction-constrained 3D molecular generation using a diffusion model enables structure-based pharmacophore modeling for drug design

**DOI:** 10.1038/s44386-026-00040-x

**Published:** 2026-03-02

**Authors:** Masami Sako, Nobuaki Yasuo, Masakazu Sekijima

**Affiliations:** 1 Department of Computer Science, Institute of Science Tokyo, Yokohama, Kanagawa Japan; 2 School of Materials and Chemical Technology, Institute of Science Tokyo, Meguro-ku, Tokyo Japan

**Keywords:** Chemistry, Computational biology and bioinformatics, Drug discovery

## Abstract

A key challenge in structure-based drug design is generating three-dimensional molecules while preserving essential protein-ligand interactions. We propose DiffPharma, a structure-based pharmacophore modeling framework based on a conditional diffusion model, to generate molecules that satisfy specified interaction constraints. The proposed method incorporates a semantic fusion architecture that integrates multiple interaction-specific neural networks, each designed to capture distinct molecular interactions such as hydrogen bonds and hydrophobic interactions. Experimental results demonstrate that DiffPharma achieves a residue-level interaction similarity of up to 0.9, significantly outperforming baseline models. To assess the method’s generalizability, ligands were generated for AKT serine/threonine kinase 1 and serine *β*-lactamase, successfully preserving key interaction features. The effectiveness of the method is demonstrated through a practical case study targeting the SARS-CoV-2 main protease. Molecular dynamics simulations indicate that the generated molecules maintain both structural stability and key interactions comparable to those of a bioactive reference ligand. In addition, the molecular mechanics generalized Born surface area (MM/GBSA) calculations based on MD trajectories suggest that several generated molecules may exhibit relatively favorable binding tendencies compared with the reference. The implementation of the DiffPharma, including code and an execution environment on Google Colab, is available under the MIT license at GitHub: https://github.com/sekijima-lab/DiffPharma.

## Introduction

Structure-based drug design (SBDD) is an important approach for rationally designing pharmacologically active molecules based on the three-dimensional structures of target proteins, and it has attracted attention since the 1980s^1–4^. In particular, a pharmacophore modeling method has been used to reproduce important interaction modes and screen molecules by extracting functional group properties that are common to active compounds with three-dimensional configurations^5–7^.

A pharmacophore represents a generalized representation of the key steric and electronic features, such as hydrogen-bond donors or acceptors, hydrophobic regions, aromatic moieties, and charged centers, that are required for a ligand to bind effectively to a target protein. When protein-ligand complexes are analyzed in terms of these features, the resulting interactions are realized through specific noncovalent contacts, including hydrogen bonds, hydrophobic interactions, *π*–*π* stacking interactions, cation-*π* interactions, salt bridges, halogen bond, and so on. Among these interactions, hydrogen bonds and hydrophobic interactions have been studied and explicitly represented in pharmacophore models and structure-based drug design studies^8,9^. Consistently, an analysis of the CrossDocked2020 dataset^10^ (Supplementary Fig. 1 and Supplementary Table 2) shows that hydrogen bonds and hydrophobic interactions are present in 94% and 84% of protein-ligand pairs, respectively, whereas other interaction types appear far less frequently.

Pharmacophore modeling has contributed to discovery of actual drug candidates in various therapeutic areas. For example, a dynamic pharmacophore modeling was used to successfully identify HIV-1 integrase inhibitors with low micromolar activity^11^, and a combined pharmacophore and docking-based approach enabled the discovery of BACE1 inhibitors for treating Alzheimer’s disease^12^. Recently, during the COVID-19 pandemic, several studies involving pharmacophore modeling approaches contributed to the rapid discovery and experimental validation of an inhibitor for the SARS-CoV-2 main protease (M^pro^)^13–15^.

As in the examples above, pharmacophore modeling has been used as an effective method for in silico screening^16,17^ by making use of large-scale databases^18–23^. For example, the ZINC-22 database contains more than 37 billion compounds^24^, and more than 100 million unique compounds can be found in the PubChem database^25^. On the other hand, the total number of drug-like molecules is estimated to be 10^11^ by GDB-17^26^, indicating that the current database screening methods can explore only a small part of the large chemical space. The limited coverage of the current databases makes it necessary to explore diverse molecular candidates beyond those that are available in the existing libraries.

In parallel with database-driven screening approaches, recent end-to-end structure prediction frameworks, such as AlphaFold3^27^ and Boltz-2^28^, have shown remarkable progress in predicting three-dimensional protein-ligand binding poses directly from protein sequence information and ligand representation. These methods implicitly learn interactions between proteins and ligand molecules from large-scale data and enable accurate pose prediction and, in some cases, binding affinity estimation.

In recent years, machine learning-based generative molecular design models have attracted attention as effective methods for tackling vast unexplored chemical spaces^29–31^. In particular, research on generating molecules directly within the ligand binding sites of proteins has emerged as a promising strategy for achieving structure-based drug discovery^32–38^. In an early attempt, Ragoza et al.^32^ utilized variational autoencoders (VAEs) to generate ligand molecules through density map prediction. Liu et al.^33^ modeled the probabilities of atoms incorporating distance and angle embeddings with a graph neural network and sequentially sampled new atoms via an autoregressive model. The characteristics of the molecule generation process implemented by an autoregressive model are that the model is strongly influenced by the initially generated structure and that the sequential sampling algorithm does not consider global information while it strongly influences local information. In contrast, diffusion model-based approaches^36–39^ perform one-shot molecular generation for all atoms in parallel, allowing the employed model to effectively incorporate the global structural information of protein pocket features.

Although these studies have succeeded in incorporating the three-dimensional structural information of proteins, they implicitly integrated the interactions between the protein and ligand molecules that are essential for drug discovery, resulting in insufficient interaction reproducibility. Recent studies have explicitly incorporated protein-ligand interactions^40,41^, however, these approaches have several limitations. Wonho et al.^40^ employed a combination of VAEs and autoregressive models that sequentially generate ligand molecules atom-by-atom, but do not fully capture spatial features such as directionality and distances crucial for hydrogen bonds. Sako et al.^41^ proposed a conditional diffusion model that represents interactions as interaction particles, but focused only on hydrogen bonds. These existing models treat different types of interactions uniformly and then do not fully exploit each of their characteristics.

In this work, we propose DiffPharma, a structure-based pharmacophore modeling framework based on a conditional diffusion model for 3D molecular generation. DiffPharma enables systematic exploration of ligand scaffolds and related chemical series under specified protein-ligand interaction constraints. By conditioning molecular generation on predefined interaction patterns, DiffPharma supports interaction-aware scaffold hopping by directly generating molecules in three dimensions within the target protein pocket.

Following the interaction statistics described above, DiffPharma focuses on hydrogen bond and hydrophobic interactions in its interaction conditioning. These interactions are represented using an interaction particle formulation, allowing hydrogen bond and hydrophobic interactions to be explicitly modeled in three dimensions with their spatial positions and orientations. Dedicated neural networks of DiffPharma are designed to extract the distinct features of the ligand binding sites of the target proteins and each type of interaction. These networks are semantically integrated through a hierarchical architecture to enable the production of accurate molecular designs. The proposed architecture allows for the effective extraction of interaction-specific features through separate processing schemes while maintaining a coherent integration strategy that balances multiple interaction types to design realistic molecules.

Evaluation results show that DiffPharma generates molecules that successfully preserve the reference pharmacophore features while maintaining structural diversity, suggesting practical applicability across diverse target proteins. To assess the generalizability of the method, ligand molecules are generated for two distinct target proteins, i.e., AKT serine/threonine kinase 1 (PDB ID: 3CQW) and serine *β*-lactamase (PDB ID: 1L2S), with the generated molecules successfully preserving key interaction patterns of the reference ligands. Furthermore, to demonstrate the practical applicability of the proposed method to drug discovery, a case study is conducted on the SARS-CoV-2 M^pro^, a critical antiviral target. Molecular dynamics (MD) simulations demonstrate that the generated molecules have comparable stability to that of the cocrystal ligand, and molecular mechanics generalized Born surface area (MM/GBSA) calculations based on MD trajectories suggest that some of the generated molecules exhibit relatively favorable binding free energy estimates compared with the reference molecule. Notably, the proposed method successfully reproduced the ligand molecule that is not included in the training data, based solely on the target protein structure and specified interaction constraints. In addition, ADMET profiling confirms that the generated molecules exhibit favorable drug-likeness and pharmacokinetic properties. It should be noted that downstream analyses such as MD simulations and ADMET-related assessments, among others, are not components of the DiffPharma model itself, but are applied as post-generation evaluation steps to assess the plausibility and quality of the generated molecules.

## Results

### Overview of the DiffPharma model

**Fig. 1:**
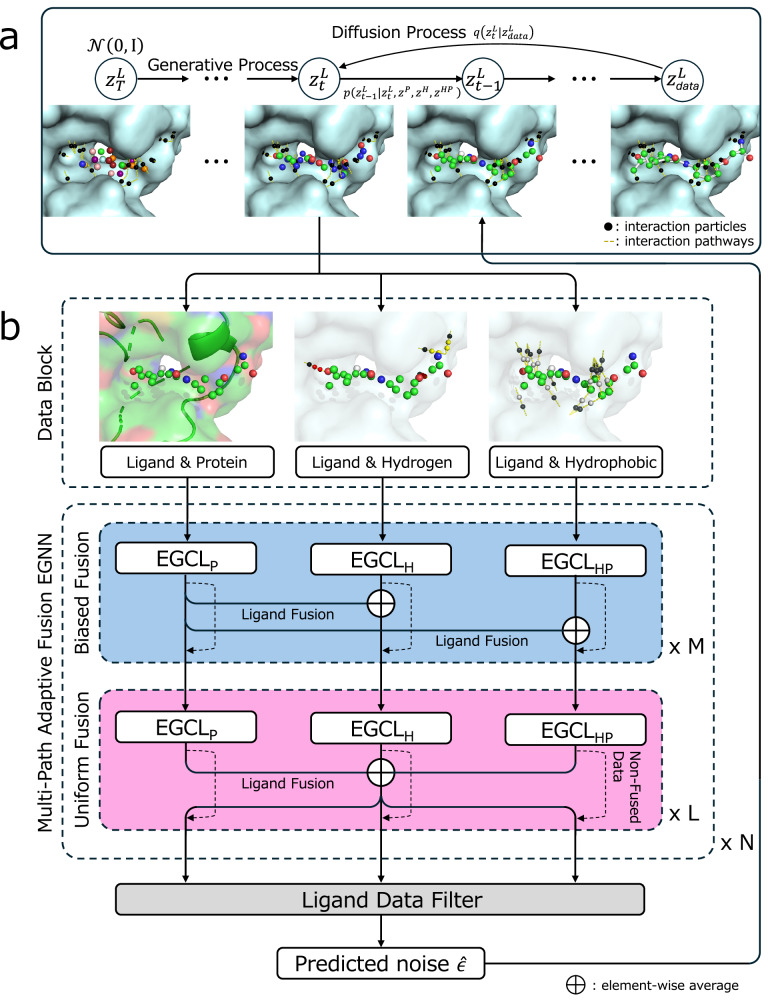
Overview of the DiffPharma framework. **a** Diagram of diffusion process of DiffPharma, which is based on the conditional diffusion model. **b** Mechanism of the denoising process for a single time step. Three types of data, i.e., ligand-protein, ligand-hydrogen interactions, and ligand-hydrophobic interactions, are created in a data block. A multi-path adaptive fusion EGNN (MAP-EGNN) is used to process the each data and to predict the noise of the denoising process. Each type of data is extracted via dedicated E(3)-equivariant graph convolutional layers (EGCL) and then only the ligand features integrated using two mechanisms: A biased fusion and a uniform fusion. In the biased fusion block, ligand-protein information complements the other two types of information, whereas in the uniform fusion block, all the information is integrated. In both fusion blocks, the ligand features are fused via element-wise averaging whereas the other features are not integrated and passed to the next layer as non-fused data. Figures generated using PyMol^67^.

DiffPharma is a conditional molecular generation model that guides the interactions between the protein and ligand molecules using a diffusion model. This model follows the denoising diffusion probabilistic model (DDPM)^42^ framework with detailed formulations provided in Section “Diffusion model formulation”. The main features of DiffPharma are (i) the explicit introduction of intermolecular interactions such as hydrogen bonds and hydrophobic interactions as “interaction particles” and their use as guidance information for performing ligand generation with the diffusion model and (ii) the a “multi-path adaptive fusion E(3)-equivariant graph neural networks (MAP-EGNN)”, which is designed to process and integrate different types of interactions, as shown in Fig. 1. The MAP-EGNN is a network equipped with multiple EGNNs^36,43^, and it maintains E(3)-equivariance similar to that of EGNNs, with the associated proof provided in Supplementary Section 1. These mechanisms enable the precise modeling of complex interaction patterns that cannot be captured by conventional methods.

The first feature of the proposed approach is that information on hydrogen bonds and hydrophobic interactions between protein and ligand molecules is explicitly introduced as “interaction particles^41^”, which guide the positions and types of particles by focusing on the spatial and chemical properties of their interactions. By placing interaction particles along the interaction pathways between the protein and ligand molecules, molecules are generated such that they naturally satisfy the shape compatibility and chemical validity properties for the binding site of the protein. Among the interaction particles, the protein-side particles control the relative positions of the protein and ligand molecules, whereas the ligand-side particles maintain the chemical validity of the ligand molecules, such as donor/acceptor information in the case of hydrogen bonds and carbon types in the case of hydrophobic interactions. A detailed explanation of this scheme is described in the Section “Interaction particle framework with anchor and functional roles”.

The second key feature consists of two components, namely, a dedicated subnetwork for each type of interaction and a mechanism that integrates their outputs. The MAP-EGNN consists of three subnetworks. The first subnetwork captures the geometric and chemical features that describe the relationship between the protein and the ligand molecules, and the second and third subnetworks handle hydrogen bonds and hydrophobic interactions, respectively.

The information acquired from these three subnetworks is then integrated by two types of fusion mechanisms (biased fusion and uniform fusion). The biased fusion selectively shares the output of the protein-ligand subnetwork with the other two subnetworks, thereby anchoring interaction-specific representations to a common protein-ligand structural context. The uniform fusion subsequently integrates the three types of interaction-aware ligand representations in a symmetric manner, allowing different interaction constraints to be jointly satisfied by preventing the ligand representation from being biased toward any single interaction type.

The final integrated information is subsequently used for denoising prediction. A comprehensive description of this component is presented in Section “Multi-path Adaptive fusion EGNN”.

### Interaction reproducibility

**Fig. 2:**
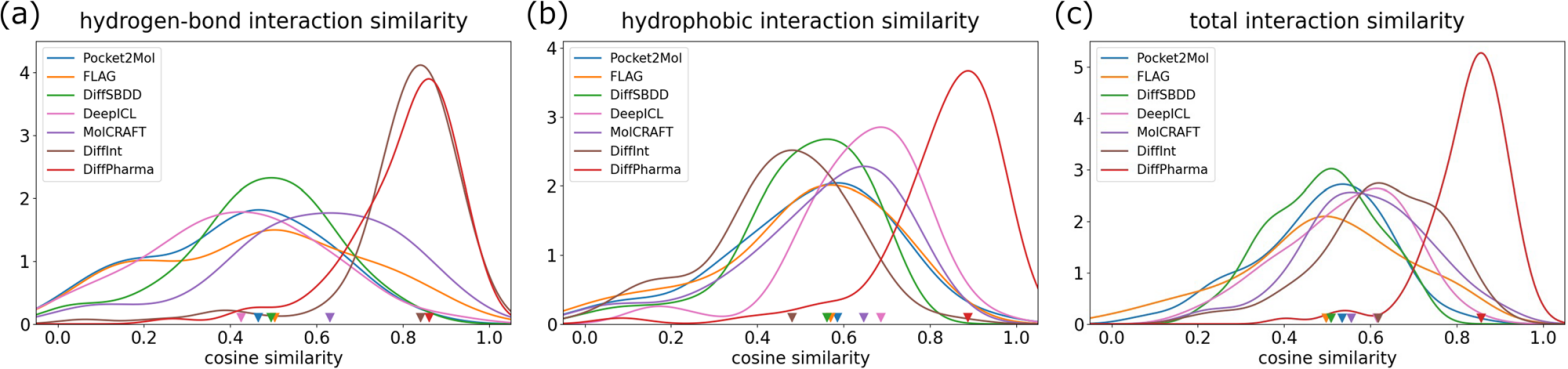
Cosine similarity–based interaction reproducibility of protein–ligand interactions across different molecular generation models. Residue-by-residue interaction patterns are compared using cosine similarity for (**a**) hydrogen-bond interactions, (**b**) hydrophobic interactions, and (**c**) total interactions. One hundred molecules are generated for each of the 100 target proteins contained in the test dataset. The residue-wise interaction pattern of each molecule is compared against that of the reference ligand. The curves represent the distribution of the average cosine similarity across all targets for each model. Downward triangles indicate the peak positions of the similarity distributions for each model. DiffPharma outperforms the other baseline models, with the total interaction similarity showing a peak around 0.9. Figures generated using Matplotlib^68^.

During the interaction-guded molecular generation process, it is important to quantitatively evaluate the extent to which the generated molecules reproduce the interaction pattern with the target protein. Interaction reproducibility is quantified using cosine similarity between residue-level interaction patterns. For each ligand-protein complex, interactions are first identified at the residue level, resulting in a binary interaction profile that indicates whether each protein residue participates in a given interaction type with the ligand. The cosine similarity between the interaction profiles of a generated molecule and the corresponding reference ligand is then computed, providing a normalized measure of how well the residue-wise interaction pattern is reproduced. The test dataset consists of 100 protein-ligand complexes, from which interaction patterns are extracted. For each protein, 100 ligand molecules are generated on the basis of protein structure and the extracted interaction information. The results of the similarity analysis conducted for hydrogen bonds, hydrophobic interactions, and both interactions are shown in Fig. 2 (a), (b), and (c), respectively. The mean and standard deviation of interaction reproducibility are summarized in Supplementary Table 3. The interaction reproducibility values for individual protein targets used to construct these distributions are provided in Supplementary Table 4 (hydrogen bonds), Supplementary Table 5 (hydrophobic interactions), and Supplementary Table 6 (total interactions). Six existing models are used as baselines to benchmark the proposed method: Pocket2Mol^33^, FLAG^34^, DiffSBDD^36^, DeepICL^40^, MolCRAFT^39^ and DiffInt^41^.

DiffPharma produces a cosine similarity distribution that peaks at approximately 0.9, indicating extremely high reproducibility of residue-level interaction patterns. Importantly, this high reproducibility is consistently observed for both hydrogen bonds and hydrophobic interactions, demonstrating that DiffPharma can accurately reproduce multiple types of protein-ligand interactions in a well-balanced manner.

To facilitate comparison, DiffPharma is evaluated together with six existing baseline models. For hydrogen bonds interactions, DiffInt achieves reproducibility comparable to that of DiffPharma, showing only slightly lower similarity. MolCRAFT attains moderate performance, whereas Pocket2Mol, FLAG, DiffSBDD, and DeepICL exhibit generally low reproducibility. For hydrophobic interactions, DeepICL and MolCRAFT perform somewhat better than the other baseline models, while Pocket2Mol, FLAG, DiffSBDD, and DiffInt show relatively low similarity. For total interaction reproducibility, DiffInt and DeepICL achieve slightly higher similarity among the baseline models. This trend is reasonable because these two models explicitly incorporate interaction information during training. However, their reproducibility is biased toward specific interaction types. DiffInt performs well primarily for hydrogen bonds interactions, whereas DeepICL is relatively strong for hydrophobic interactions. As a result, neither model attains balanced reproducibility of both interaction types, and both remain substantially inferior to DiffPharma.

In addition to interaction reproducibility, the fundamental molecular properties of the generated molecules are also investigated as listed in Supplementary Table 7. The molecules generated by DiffPharma are shown to be close to the characteristic values of the test set. It is also evaluated whether interaction constraints limit the diversity of generated molecules. A target-wise analysis revealed that molecular diversity is retained across targets, even when high interaction similarity is achieved. However, a moderate trade-off is observed, where higher interaction similarity is associated with a slight decrease in diversity. A quantitative assessment of the relationship between interaction similarity and internal diversity is provided in the Supplementary Fig. 3.

An ablation study of DiffPharma shows that each interaction-specific module contributes to reproducing its respective interaction type and that their integration ensures overall balanced performance, as shown in the Supplementary Fig. 2. Additionally, *π* − *π* stacking interactions are treated as a subset of distance-based hydrophobic interactions without explicit *π* − *π* specific directional constraints, and DiffPharma shows the capability to recover such interactions to some extent, as assessed by distance and angle based interaction analysis between the generated molecules and target proteins using ODDT with detailed results in the Supplementary Section 4.4.

Furthermore, to evaluate the stereochemical validity of the generated molecules, the KL divergence values for bond lengths, bond angles, and dihedral angle distributions between the generated molecules and the test set are summarized in Supplementary Table 8. DiffPharma achieves comparable or even superior performance to other models in reproducing bond length (Supplementary Fig. 4) and dihedral angle distributions (Supplementary Fig. 6), while showing slightly lower agreement with the reference distributions for bond angles (Supplementary Fig. 5). These results suggest that the generated molecules by DiffPharma preserve overall stereochemical validity in addition to reproducing protein-ligand interactions. The generated poses are also evaluated using docking-based CNN scores to assess pose plausibility and relative binding tendency, as summarised in Supplementary Table 9 and in Supplementary Fig. 7.

As a complementary evaluation, the efficiency of the DiffPharma molecular generation process is characterized. Chemical validity is assessed by checking whether the generated molecules could be successfully parsed as valid molecular structures using RDKit. Using this criterion, 9,607 out of 10,000 generated molecules (96.1%) are chemically valid as reported in Supplementary Section 5.2. The generation of 10,000 molecules required 24,730 seconds on a single NVIDIA H100 GPU using the TSUBAME4.0 supercomputer at the Tokyo Institute of Science.

Overall, these comprehensive evaluation results confirm that DiffPharma accurately reproduces the given interaction patterns, demonstrating its effectiveness as a interaction-guded molecular generated model.

### Evaluating the chemical feasibility of interaction-guided molecular generation

**Fig. 3:**
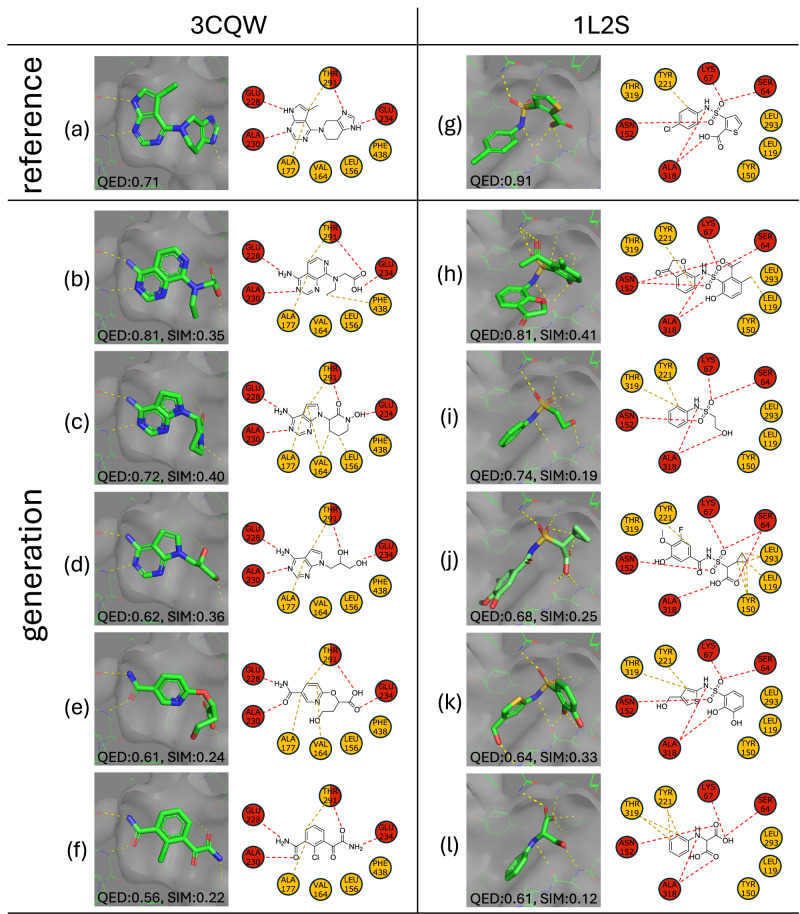
The 3D poses and interaction patterns of ligand molecules with the target proteins (PDB IDs: 3CQW and 1L2S). **a**, **g** Reference ligands for 3CQW and 1L2S, respectively. **b**–**f** and **h**–**l** The molecules generated for 3CQW and aL2S, respectively. The five generated molecules for each target protein are displayed in descending QED order; those that satisfy all the interaction conditions used during the generation process and for which synthetic routes are identified through retrosynthetic analysis are selected through screening. Each molecule is shown with its QED score and its Tanimoto similarity^69^ relative to the reference molecule, wich are calculated using the RDKit fingerprint^70^. Figures generated using PyMol.

To assess the generalizability of DiffPharma, ligand molecules are generated for two additional drug discovery targets which are included in PDB^18^, including AKT serine/threonine kinase 1 (PDB ID: 3CQW) and serine *β*-lactamase (PDB ID: 1L2S) under predefined interaction constraints. This experiment evaluates the ability of the generated molecules to preserve the key protein-ligand interaction patterns of the reference ligands while maintaining structural diversity. In addition, example retrosynthetic analyses are presented for selected generated molecules. The original ligand molecules of 3CQW and 1L2S and their interactions are shown in Figs. 3(a) and (g), respectively. The reference molecule of 3CQW has hydrogen bonds with four residues (GLU28, ALA230, GLU234, and THR291) and hydrophobic interactions with two residues (ALA177 and THR291), and the reference molecule of 1L2S has hydrogen bonds with four residues (SER64, LYS67, ASN152, and ALA318) and hydrophobic interactions with one residue (TYR221).

Ligand molecules are generated on the basis of predefined interaction patterns with each target protein. The generated molecules are subjected to two screening steps. The first step involves satisfying all specified interaction constraints that are used during the generation process. The second step involves conducting a retrosynthetic analysis with SciFinder^*n*^^44^ to identify molecules with feasible synthetic routes, thereby assessing their practical stability and synthetic accessibility. Among the screened molecules, five molecules with high quantitative estimates of drug-likeness (QED)^45^ scores are selected and shown in Figs. 3(b) to (f) for 3CQW and (h) to (l) for 1L2S, along with their QED scores and similarities to the reference molecules.

The reference molecule of 3CQW has a heterocyclic compound called 7H-pyrrolo[2,3-d]pyrimidine, which is a fused ring with a 5-membered pyrrole and a 6-membered pyrimidine. This moiety forms hydrophobic interactions with THR291 and ALA177. The generated molecules in (b), (c), and (d) also contain similar heterocyclic structures, reflecting the reproduction of these hydrophobic interactions during the molecular generation process.

In the reference molecule of 1L2S, the two oxygen atoms of the sulfonyl group form hydrogen bonds with the SER64, LYS67, ASN152, and ALA318 residues, and the chlorophenyl group forms hydrophobic interactions with the TYR221 residue. Four of the generated molecules, specifically those shown in (h)-(k), each contain a secondary sulfonamide group, as found in the reference molecule (g), which reproduces the hydrogen-bond interactions. Furthermore, the reproduction of aromatic rings that form hydrophobic interactions with TYR221 is preserved as a result of the interaction constraints applied during the generation process.

**Fig. 4:**
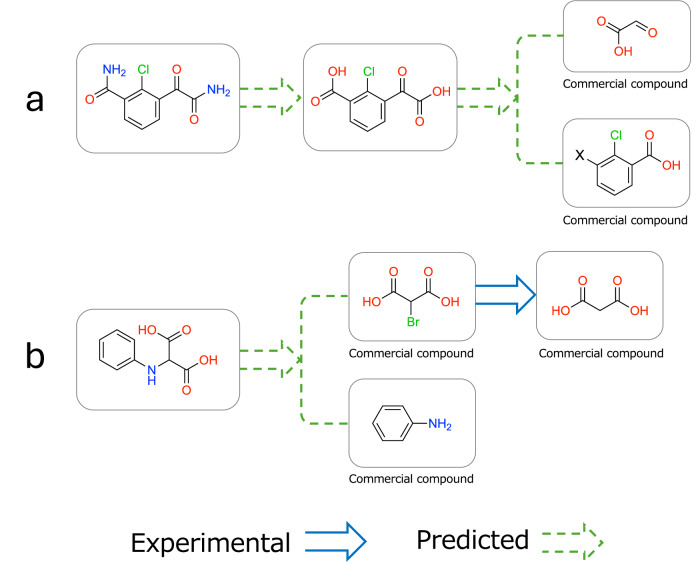
Synthetic pathway analyzed by SciFinder^*n*^^44^. **a** The Synthetic pathway for the molecule displayed in Fig. 3f. **b** The Synthetic pathway for the molecule displayed in Fig. 3l. Figures generated using ChemDraw^71^.

These results demonstrate that it is possible to generate new molecules that structurally differ from the original molecule while preserving the interactions of the reference ligand molecule. A retrosynthetic analysis of selected examples indicates that some generated molecules admit plausible synthetic routes, suggesting their chemical plausibility. As representative examples of synthetic routes, the retrosynthetic pathways for the molecules shown in Figs. 3(f) and (l) are presented in Figs. 4(a) and (b), respectively. The retrosynthetic pathways for the other generated molecules targeting 3CQW and 1L2S are provided in Supplementary Figs. 8 and 9, respectively.

Notably, the molecules shown in Fig. 3d, l have been registered in chemical databases such as PubChem, with CIDs of 92146926 and 20036755, respectively, and are known to be stable compounds. These findings support the feasibility of generating stable, synthetically tractable molecules through an interaction-guided molecular design procedure.

### A drug discovery case study using the SARS-CoV-2 M^pro^

In the drug discovery process, specific intermolecular interactions between the protein and ligand molecules play a central role in determining both the structural stability and binding affinity of the resulting complexes. Incorporating predefined interaction constraints into the molecular generation process enables more rational and efficient ligand design by guiding key pharmacophoric features. In this study, the influence of interaction constraint selectivity on the spatial distribution, dynamic stability, and binding affinity of the generated molecules is investigated.

First, the influence of interaction selectivity on the spatial distribution of the generated molecules is evaluated. It is evaluated that the probability of achieving molecular generation with selectivity for predefined interactions and the effect of this selectivity on the pose stability and binding free energy of the molecular generation process conducted within the target protein. Second, MD simulations are performed to assess the structural stability of the generated binding poses within the ligand binding site of the target protein. Third, the binding affinities of the generated molecules are estimated using MM/GBSA calculations based on MD trajectories. Fourth, the drug-likeness of the generated molecules is assessed by predicting physicochemical and ADMET-related properties using ADMETlab 3.0.

**Fig. 5:**
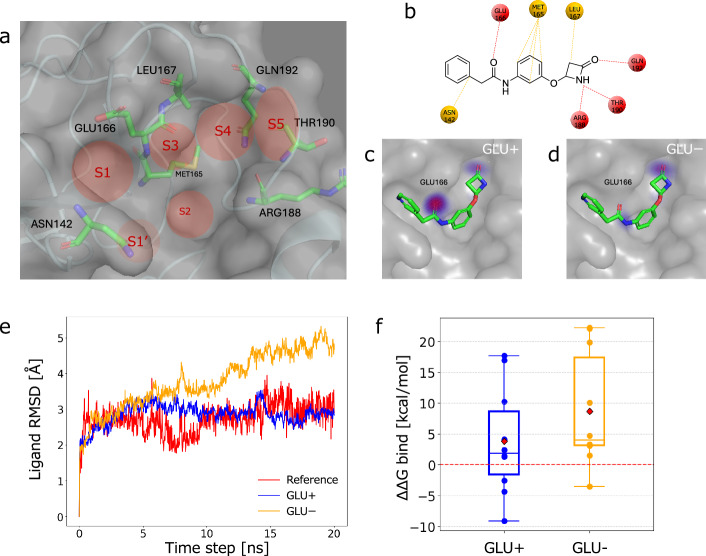
Verification results of the molecular generation process performed for M^pro^. **a** Active sites consisting of multiple sub-pockets (S1, S1', S2, S3, S4, and S5) for M^pro^. **b** Interaction pattern for PDB ID 7GBL. **c**, **d** Reference poses within the binding pocket and density distributions of the acceptor atoms in molecules generated under GLU+ and GLU- conditions, respectively. **e** Ligand RMSDs derived from MD simulations of the reference molecule and the average values for molecules generated under GLU+ and GLU- conditions. **f** Binding free energy for the molecules generated under GLU+ and GLU- conditions. The dashed line shows the binding free energy for the reference molecule. Figures generated using PyMol and Maestro^72^.

The SARS-CoV-2 M^pro^ is selected as a case study involving a representative drug discovery target. While many inhibitors designed thus far have been covalent inhibitors that form covalent bonds with cysteine residues^46–49^, recent attention has been focused on noncovalent inhibitors that exhibit high inhibitory activity through multiple noncovalent interactions^50–53^. M^pro^ has active sites consisting of multiple subpockets (S1, S2, S3, S4, and S5) as shown in Fig. 5a, and the following characteristic interactions with ligand molecules have been observed in each subpocket: hydrogen bonds with GLU166 and HIS163 and hydrophobic interactions with ASN142 in the S1 subpocket, *π* − *π* stacking with HIS41 and hydrophobic interactions with MET165 in the S2 subpocket, and hydrogen bonds with GLU192 and GLN189 in the S3,4,5 subpockets^54,55^.

The cocrystal structure of a noncovalent inhibitor binding to M^pro^ (PDB ID: 7GBL) is used as a reference. The ligand and water molecules are removed from the reference data, and the resulting protein structure is used as the target protein for molecular generation process. The ligand is subsequently redocked into the binding site using Vina-GPU+^56^, and the corresponding interaction pattern is subsequently determined through visual inspection, as shown in Fig. 5b. The redocked inhibitor forms hydrogen bonds with GLU166 in the S1 subpocket, and with ARG188, THR190, and GLN192 in the S3, S4, and S5 subpockets, respectively, as well as hydrophobic interactions with ASN142 in S1, MET165 in S2, and LEU167 in S3. These interactions are used as the interaction constraints for the molecular generation process.

Among these interactions, the hydrogen bond with GLU166, which is formed via the amido group of the reference molecule, is selected to clarify the influence of interaction selectivity on the molecular design process. Specifically, molecular generation is conducted under two distinct settings, where one incorporates all interactions including the hydrogen bond with GLU166 (GLU+), whereas the other specifically excludes GLU166 (GLU-).

Notably, the interaction pattern extracted from the 7GBL complex does not appear in the training data. Therefore, the M^pro^ case study evaluates DiffPharma on a previously unseen interaction specification. Additional details regarding the training data and sequence similarity analysis are provided in the Supplementary Section 7.

The influence of interaction constraint selectivity is evaluated by analyzing the spatial distributions of generated molecules, focusing on acceptor atom localization under the GLU+ and GLU- conditions. Figure 5c–d shows the density distributions of the acceptor atoms among the 10,000 molecules generated under each condition. In the S4 subpocket, both the GLU+ and GLU- conditions result in consistent concentrations of acceptor atoms. In contrast, around the S1 subpocket, the GLU+ condition yields a high-density localization of acceptor atoms around GLU166, whereas the GLU- condition produces a broader distribution with no distinct concentration around GLU166.

This result indicates that even under the GLU- condition, the properties of the protein residues may implicitly guide the placement of atoms that are relevant to hydrogen bond formation. However, since hydrogen bonds require strict geometrical constraints such as distance and orientation constraints, such implicit information appears insufficient for guiding acceptor atoms to the appropriate positions that are required for hydrogen bonds. On the other hand, under the GLU+ condition, the explicit hydrogen-bond conditions with GLU166 places the acceptor atoms precisely in their appropriate positions and orientations, indicating a higher probability of guidance toward the appropriate geometrical arrangement.

These results show differences in static atomic distributions between the two conditions, followed by an evaluation of their dynamic stability. MD simulations are performed using the Desmond module in Maestro (Schrödinger Suite 2024-1, D. E. Shaw Research)^57^ to verify whether the molecules generated under these interaction conditions form stable complexes with the binding site of the target protein. From the generated molecules, those satisfying all interaction constraints used during generation are extracted. To ensure equal screening between the two conditions, the hydrogen-bond constraint with GLU166 is excluded from the screening process. As a result, 251 and 243 ligand molecules are obtained for the GLU+ and GLU- conditions, respectively. In addition, the reproducibility of hydrogen-bond and hydrophobic interactions under each condition is analyzed at the residue level (Supplementary Fig. 10). For each of the GLU+ and GLU- conditions, the ten ligand molecules with the highest QED values are selected from the screening results and subjected to MD simulations. The detailed settings employed for MD simulations are described in Supplementary Section 8.

A trajectory analysis of the ligand poses produced during the MD simulations is used to calculate root-mean-square deviations (RMSDs). The average RMSDs determined across the ten selected ligand molecules for each condition are presented in Fig. 5e. The RMSDs of the ligand molecules generated under the GLU+ condition remain comparable to those of the reference ligand in 7GBL, indicating the high structural stability of the ligand-protein complexes. In contrast, the molecules generated under the GLU- condition exhibit increasing RMSDs over time, suggesting deviations from the binding pocket. This behavior is likely due to the absence of a hydrogen-bond condition with GLU166, resulting in reduced binding stability. In addition to the averaged RMSDs, the individual MD trajectories of the ten selected ligands under both GLU+ and GLU- conditions, yielding a total of 20 trajectories, are provided in Supplementary Figs. 11–14.

To thermodynamically evaluate the stability of each ligand-protein complex obtained from the MD simulations, MM/GBSA calculations are performed using the Prime module (Schrödinger Suite 2024-1, Schrödinger, LLC)^58^. The calculations use the final 100 snapshots (2 ns) of each trajectory. Binding free energies Δ*G*_*bind*_ are then converted to relative binding free energies, ΔΔ*G*_*bind*_, defined as ΔΔ*G*_*bind*_ = Δ*G*_*bind*_ − Δ*G*_*bind*_(*ref*), with the binding free energy of the reference ligand given by Δ*G*_*bind*_(*ref*)= -52 kcal/mol. The distributions of ΔΔ*G*_*bind*_ values for ten generated molecules per condition under the GLU+ and GLU- settings are shown in Fig. 5f. The average ΔΔ*G*_*bind*_ under the GLU+ condition is 3.8 kcal/mol, which is lower than the corresponding value of 8.7 kcal/mol observed under the GLU- condition, suggesting a relatively more favorable binding tendency under the GLU+ condition within the MM/GBSA analysis. Furthermore, three molecules generated under the GLU+ condition exhibit negative ΔΔ*G*_*bind*_ values, indicating that their relative binding free energy estimated by MM/GBSA are more favorable than that of the reference ligand.

**Fig. 6:**
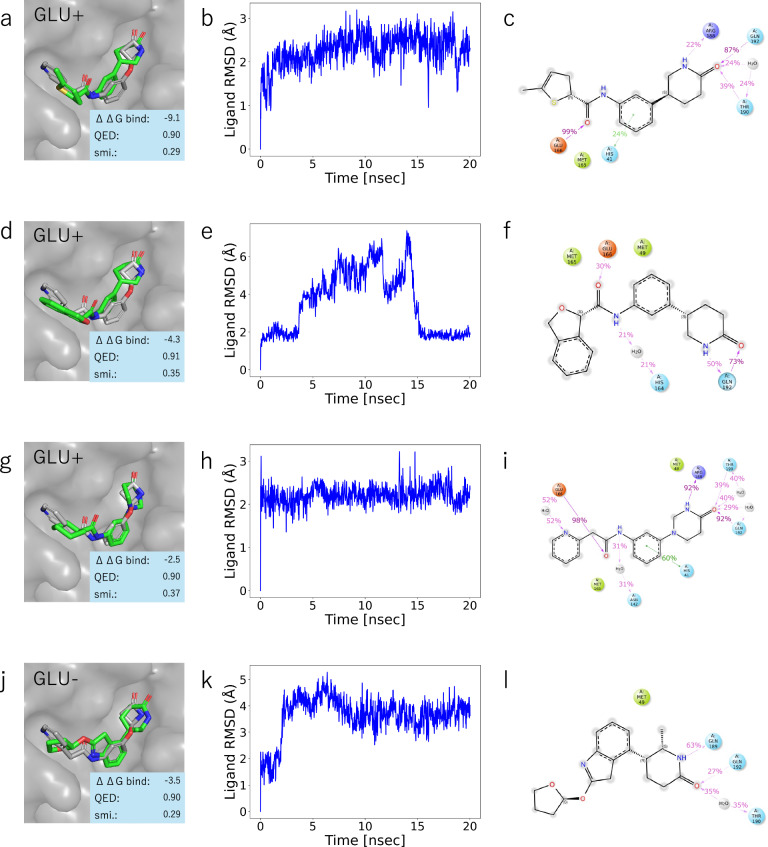
MD analysis of selected generated molecules with negative ΔΔ*G*_*bind*_ relative to the reference ligand. The top three rows correspond to the GLU+ conditions (top to bottom, ordered by ΔΔ*G*_*bind*_) and the bottom row correspond to the GLU- condition. For each molecule, panels show (**a**, **d**, **g**, **j**) an overlay of the generated molecule (green) and the reference ligand (gray) in the binding pocket, (**b**, **e**, **h**, **k**) ligand RMSD during a 20 ns MD simulation, and (**c**, **f**, **i**, **l**) the 2D ligand representation with interaction frequencies observed during MD. Figures generated using PyMol, Matplotlib and Maestro.

Among the generated molecules under each condition, those exhibiting improved relative binding free energies (ΔΔ*G*_*bind*_ < 0) are selected for further analysis. As a result, three molecules are identified under the GLU+ condition, whereas one molecule satisfies this criterion under the GLU- condition. For these selected molecules, the generated poses, the ligand RMSD trajectories obtained from the MD simulations, and the interaction occupancies observed during the MD simulations are shown in Fig. 6. The three GLU+ molecules are ordered from most to least favorable based on (ΔΔ*G*_*bind*_ < 0), followed by the subsequent GLU- case. The GLU+ ligands stably maintain the hydrogen bond with GLU166 throughout the MD simulations, whereas the GLU- ligand does not maintain this interaction. Although the interaction occupancy with GLU166 is reduced for the second and third-ranked GLU+ molecules compared to the top-ranked one, the hydrogen bond remains persistently formed during the simulations. Moreover, the GLU+ ligands also have relatively high interaction occupancies for other residues, as shown in Supplementary Fig. 15. These results are consistent with those of previous studies^59,60^, indicating that the formation of stable interactions throughout MD simulations tends to result in more favorable binding free energies in MM/GBSA calculations.

These results demonstrate that the presence or absence of specific interaction constraints influences not only the static arrangement of interactions but also the structural stability observed during MD simulations and the relative binding free energy trends estimated by MM/GBSA.

**Fig. 7:**
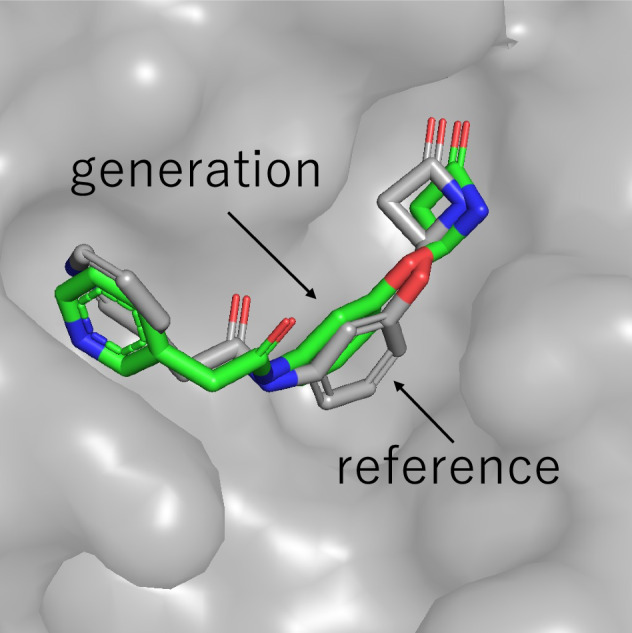
Superimposed 3D binding poses of the reference ligand (PDB ID: 7GBL) and the generated molecule within the binding pocket of M^pro^. The reference ligand (gray) and the generated molecule (green) show highly consistent spatial alignment. Figures generated using PyMol.

In addition to generating diverse ligand candidates, the proposed method also succeeds in reproducing a known bioactive compound. A molecule identical to the reference inhibitor used in this study (PDB ID: 7GBL), which is not included in the training data, is de novo generated solely based on the target protein structure and predefined interaction constraints without any knowledge of the reference compound itself. This reference compound has been experimentally validated to exhibit inhibitory activity against M^pro^ (IC_50_ = 88.3 μM) and the generated molecule adopts a highly similar 3D binding pose to that of the reference inhibitor as shown in Fig. 7. This result demonstrates that DiffPharma can generate pharmacologically relevant molecules that capture the essential interaction patterns encoded in the input constraints, indicating its potential as a structure-based molecular design framework.

**Fig. 8:**
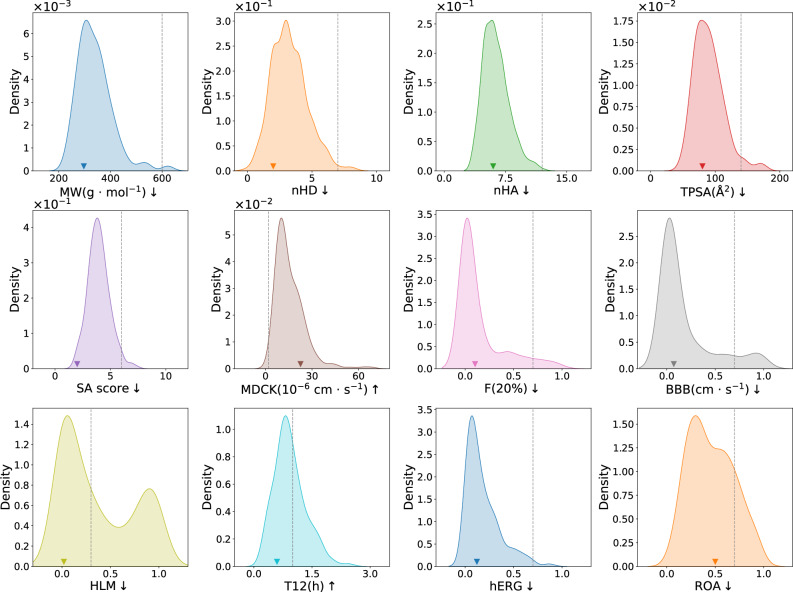
Distributions of ADMET properties. Properties are categorized into physicochemical properties (MW, nHD, nHA, TPSA), medicinal chemistry property (SA score), and ADMET properties, which include absorption (MDCK, F(20%)), distribution (BBB), metabolism (HLM), excretion (T12), and toxicity (hERG, ROA), and are calculated using ADMETlab 3.0. Dashed lines indicate threshold values defined by ADMETlab 3.0 and arrows next to each property label show the preferred direction relative to these thresholds. Molecular weight (MW) has an optimized value of 100-600 g/mol. The number of hydrogen-bond donors (nHD) and acceptors (nHA) are preferred in ranges of 0-7 and 0-12 respectively. Synthetic accessibility score (SA score) below 6 is considered favorable for ease of synthesis. Topological polar surface area (TPSA) between 0-140 Å^2^ is optimal. Madin-Darby Canine Kidney cells permeability (MDCK) represent an in vitro model for permeability screening and values greater than 2 × 10^−6^ cm/s predict efficient drug absorption. The human oral bioavailability (F(20%)) predicts the efficiency of the drug delivery to the systemic circulation and is preferably less than 0.7. Blood-brain barrier penetration (BBB) predicts the probability that a drug can cross the blood-brain barrier and reach its molecular targets in the central nervous system and values below 0.7 are desirable. human liver microsomal stability (HLM) estimates the potential for hepatic clearance and values below 0.7 are desirable. T12 is the half-life of a drug, which reflects both clearance and volume of distribution, with 1 hour or longer being considered desirable. hERG is an indicator that evaluates the risk of blocking cardiac potassium channels involved in repolarization, which may lead to serious side effects such as arrhythmia and sudden death and values below 0.7 are desirable. Rat Oral Acute Toxicity (ROA) evaluates the risk of acute toxicity in mammals such as rats or mice and values below 0.7 are desirable. Figures generated using Matplotlib.

Finally, in addition to the previously evaluated binding characteristics, the drug-likeness and practical applicability of the generated molecules are assessed to evaluate their suitability for drug discovery. ADMET-related descriptors are predicted for 251 GLU+ molecules that are selected based on interaction constraint screening, excluding the hydrogen bond with GLU166. The predicted properties include physicochemical descriptors, medicinal chemistry scores, and ADMET properties (Absorption, Distribution, Metabolism, Excretion, Toxicology) as shown in Fig. 8. Most of the molecules generated by DiffPharma satisfy the criteria indicated by ADMETLab3.0^61^ for drug-likeness.

## Discussion

We propose a structure-based pharmacophore modeling framework based on a conditional diffusion model for generating 3D molecules: DiffPharma. Interaction particles are introduced to represent multiple types of molecular interactions, along with anchor particles that guide the relative positions of the protein and ligand molecules to provide increased spatial accuracy. Interaction-specific features are further captured by dedicated neural networks that are specialized for each interaction type and semantically integrated through the MAP-EGNN architecture.

First, the proposed method achieves the highest interaction pattern reproducibility among all baseline models in a benchmark of 100 protein-ligand complexes, with performance evaluated by cosine similarity. Second, the generalizability of the proposed method are demonstrated through the ligand molecules designed for two drug targets, AKT serine/threonine kinase 1 and serine *β*-lactamase. The generated molecules successfully preserve the key interaction patterns of the reference ligands while maintaining structural diversity. Finally, as a practical case study in drug discovery, a diverse set of inhibitors targeting the SARS-CoV-2 M^pro^ are designed and computationally evaluated for their binding properties. The selective application of interaction constraints enables control over the spatial pharmacophore characteristics of the generated molecules. The generated molecules are subsequently evaluated through MD simulations, exhibiting binding pose stability comparable to that of a known bioactive reference ligand, and through MM/GBSA calculations based on MD trajectories, which suggest favorable binding free energy trends for several compounds compared with the reference ligand. The reproduction of the experimentally validated reference molecule based solely on protein structure and interaction constraints indicates the practical applicability of this approach. In addition, ADMET profiling indicated that the generated molecules possess favorable drug-likeness and pharmacokinetic properties, supporting their potential applicability in drug discovery.

Although DiffPharma reproduces the average molecular property values of the test set, including QED and logP, it does not incorporate modules specifically designed to optimize such physicochemical properties, with details listed in the Supplementary Section 5.1. Future work will explore multi-objective optimization strategies to simultaneously control pharmacophore interactions and molecular properties.

DiffPharma focuses on modeling hydrogen bond and hydrophobic interactions, whereas protein-ligand binding also involves other interaction types such as *π*–*π* stacking interactions, cation-*π* interactions, salt bridges,halogen bond, water-mediated contacts, and so on. The MAP-EGNN architecture is designed to be extensible and can accommodate additional interaction representations in future extensions.

This study highlights a promising direction for accelerating the discovery of future drugs through an interaction-guided molecular design scheme.

## Methods

### Graph-based input representation

DiffPharma represents all input data in a graph-based form, where the graphs of ligand molecules, proteins, interaction particles for hydrogen bonds and hydrophobic interactions are represented as ${{\mathcal{G}}}^{L}={\{({x}_{{L}_{i}},{h}_{{L}_{i}})\}}_{i=1}^{{N}_{L}}$, ${{\mathcal{G}}}^{P}={\{({x}_{{P}_{i}},{h}_{{P}_{i}})\}}_{i=1}^{{N}_{P}}$, ${{\mathcal{G}}}^{H}={\{({x}_{{H}_{i}},{h}_{{H}_{i}})\}}_{i=1}^{{N}_{H}}$, and ${{\mathcal{G}}}^{HP}={\{({x}_{H{P}_{i}},{h}_{H{P}_{i}})\}}_{i=1}^{{N}_{HP}}$, respectively. Here, ${x}_{i}\in {{\mathbb{R}}}^{3}$ denotes the three-dimensional Cartesian coordinate vector, and *h*_*i*_ represents a one-hot vector for each particle type as listed in Table 1

**Table 1:** Node feature definitions used in DiffPharma for ligands, proteins, hydrogen particles, and hydrophobic particles

Notation	Node Category	Possible Values
${h}_{{L}_{i}}$	Ligand atom feature	C, N, O, F, P, S, Cl, Br, I, B, else
${h}_{{P}_{i}}$	Protein atom feature	C, N, O, F, P, S, Cl, Br, I, B, else
${h}_{{H}_{i}}$	Hydrogen atom feature	anchor, donor, acceptor
${h}_{{H}_{i}}$	Hydrophobic atom feature	anchor, aromatic C, sp C, sp^2^ C(not aromatic), sp^3^ C, else

In the data block shown in Fig. 1b, to capture the relationships between the ligand data and other types of data, three types of pairwise data blocks are constructed: $({{\mathcal{G}}}^{L},{{\mathcal{G}}}^{P})$, $({{\mathcal{G}}}^{L},{{\mathcal{G}}}^{H})$, and $({{\mathcal{G}}}^{L},{{\mathcal{G}}}^{HP})$. An adjacency matrix *a*_*ij*_ between all the nodes in each pairwise data is constructed based on the edge cutoff distances defined in Supplementary Table 2. Through the EGNN modules of DiffPharma, these graph-based representations enable the extraction and integration of ligand, protein, and interaction particle information.

### Interaction particle framework with anchor and functional roles

The interaction particle model^41^ places particles along predefined interaction paths to represent intermolecular interactions. In this study, the model is extended to more precisely capture both the spatial orientations and chemical properties of these interactions. Each interaction path is divided into multiple segments, and interaction particles are placed at the dividing points, with each particle assigned specific roles.

The particles that are located closest to the protein are defined as “anchor particles”, which play an important role in controlling the relative positional relationship between the protein and ligand molecules. In contrast, the particles positioned on the ligand molecule side are defined as “functional particles”, which encode the interaction pattern and appropriately guide the ligand atoms to satisfy the predefined pharmacophore.

**Fig. 9:**
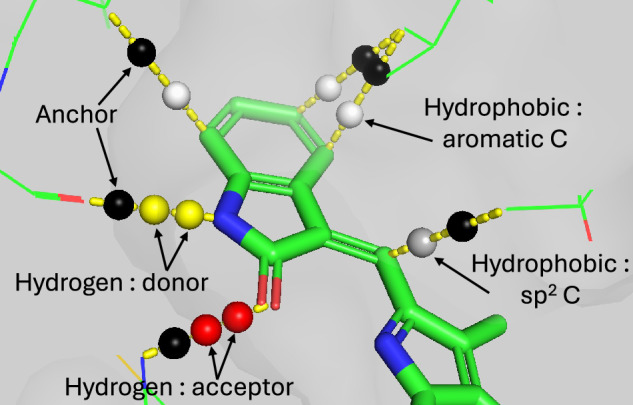
Interaction particles placed along the interaction lines (yellow dashed lines), representing hydrogen bond and hydrophobic interactions. Black spheres are anchor particles that define the relative position between the protein and ligand, and play a common role in both hydrogen bond and hydrophobic interactions. Red and yellow spheres indicate ligand atoms involved in hydrogen bond, denoting electron acceptors and donors, respectively. White and gray spheres correspond to hydrophobic interactions, indicating aromatic carbon and sp^2^ carbon atoms on the ligand, respectively. Only a subset of interaction particles is shown for clarity. Figures generated using PyMol.

The interaction pathway of a hydrogen-bond interaction is divided into four equal segments to yield three intermediate points. The points that are closest to the protein side are assigned anchor particles, whereas the remaining two functional particles provide information on which hydrogen bond is a donor or acceptor as shown in Fig. 9. On the other hand, the interaction pathway of the hydrophobic interaction is divided into three equal parts to yield two intermediate points. Following a similar approach as that employed for hydrogen bonds, the protein-side particles are assigned anchor particles, whereas the functional particles on the ligand side encode information about the particle types (e.g., aromatic, sp, sp^2^, and sp^3^ carbon), as shown in Fig. 9. The corresponding node types and their feature definitions are summarized in Table 1. This classification scheme leads to accurately characterized hydrophobic interactions.

Thus, the proposed framework introduces a unified concept of “anchor particles” across different interaction types while assigning functional particles on the basis of the specific chemical characteristics and spatial patterns of each interaction.

### Multi-path adaptive fusion EGNN

We propose a MAP-EGNN to effectively handle different types of molecular interactions as shown in Fig. 1. The architecture consists of three equivariant graph convolutional layers (EGCLs: EGCL_P_, EGCL_H_, and EGCL_HP_), with each network dedicated to specific interactions.

EGCL_P_ focuses on the relationship between the protein and ligand molecules, capturing the overall molecular shape and chemical properties of the ligand molecules. EGCL_H_ specializes in addressing the relationships between the interaction particles of hydrogen bonds and ligand molecules, enabling the production of accurate representations of distances and bond angles. EGCL_HP_ handles the relationships between the interaction particles of hydrophobic interactions and ligand molecules, learning interaction patterns that are suited for hydrophobic sites.

These specialized EGCLs do not operate independently but instead integrate with each other through two specialized fusion mechanisms, namely the bias fusion block and the uniform fusion block. These two fusion mechanisms are designed to play complementary roles. The biased fusion introduces protein-ligand structural information as a shared geometric reference into interaction-specific pathways, whereas the uniform fusion symmetrically aggregates the interaction-aware ligand representations to prevent bias toward any single interaction type. Whereas the ligand representations are fused through these blocks, the representations of the proteins, hydrogen bonds, and hydrophobic interactions are preserved as non-fused data and passed to the next module without integration.

In the biased fusion block, protein-ligand information is propagated to each of the processing pathways for hydrogen bonds and hydrophobic interactions as follows:

1 $$\begin{array}{r}{x}_{L}^{t+1},{h}_{L}^{t+1}=\left\{\begin{array}{ll}EGC{L}_{P}({x}_{L}^{t},{h}_{L}^{t},{x}_{P}^{t},{h}_{P}^{t}) & \,{\rm{for\; ligand\; -\; pocket\; data}}\\ (EGC{L}_{H}({x}_{L}^{t},{h}_{L}^{t},{x}_{H}^{t},{h}_{H}^{t})+EGC{L}_{P}({x}_{L}^{t},{h}_{L}^{t},{x}_{P}^{t},{h}_{P}^{t}))/2 & \,{\rm{for\; ligand\; -\; hydrogen\; data}}\\ (EGC{L}_{HP}({x}_{L}^{t},{h}_{L}^{t},{x}_{HP}^{t},{h}_{HP}^{t})+EGC{L}_{P}({x}_{L}^{t},{h}_{L}^{t},{x}_{P}^{t},{h}_{P}^{t}))/2 & \,{\rm{for\; ligand\; -\; hydrophobic\; data}}\,,\end{array}\right.\end{array}$$

leading to an overall contextual representation of the interaction by integrating the structural information of the protein into the processing steps for each interaction particle In this biased fusion, the protein-ligand pathway (EGCL_P_) is explicitly included in all fusion operations, such that interaction-specific ligand representations are updated under a shared protein-ligand structural prior.

In the uniform fusion block, the protein structure, hydrogen bond, and hydrophobic interaction information are aggregated into a single consistent representation as follows:

2 $${x}_{L}^{m+1},{h}_{L}^{m+1}=\frac{1}{3}\mathop{\sum }\limits_{t\in \{P,H,HP\}}EGC{L}_{t}({x}_{L}^{t},{h}_{L}^{t},{x}_{t}^{t},{h}_{t}^{t}).$$

This symmetric aggregation treats all interaction-aware ligand representations equally, thereby preventing bias toward any particular interaction type while enabling the joint satisfaction of multiple interaction constraints.

The MAP-EGNN, as shown in Fig. 1, is composed of N stacked modules, each consisting of L-layer biased fusion blocks followed by M-layer uniform fusion blocks.

### Diffusion model formulation

The diffusion process gradually adds a predetermined amount of Gaussian noise to the input data. The process of generating *z*_*t*_ by adding noise to *z*_*t*−1_, which is the latent noise representation at time step *t*-1, is represented as follows:

3 $$q({z}_{t}| {z}_{t-1})={\mathcal{N}}({z}_{t};\bar{{\alpha }_{t}}{z}_{t-1},{\beta }_{t}I),$$

where $\bar{{\alpha }_{t}}$ and *β*_*t*_ control the extent of the mixture between the original data and the Gaussian noise^42^. *β*_*t*_, which is known as the noise schedule, satisfies 0 < *β*_0_ < *β*_1_ < … < *β*_*T*_ < 1, and has the following relation: $\bar{{\alpha }_{t}}=\sqrt{1-{\beta }_{t}}$. The entire noise process is formulated as a Markov chain and expressed as follows:

4 $$q({z}_{1},\ldots ,{z}_{T}| x)=q({z}_{0}| x)\mathop{\prod }\limits_{i=1}^{N}q({z}_{t}| {z}_{t-1}).$$

The noise representation observed at an arbitrary step t is expressed in closed form by using the reparameterization trick:

5 $$q({z}_{t}| x)={\mathcal{N}}({z}_{t};{\alpha }_{t}x,{\sigma }_{t}^{2}{\bf{I}}),$$

where ${\alpha }_{t}={\prod }_{s=1}^{T}(1-{\beta }_{s})$ and ${\alpha }_{t}=\sqrt{1-{\sigma }_{t}^{2}}$. *α*_*t*_ is scheduled to smoothly transition from *α*_0_ ≈ 1 to *α*_*T*_ ≈ 0, and for a sufficiently large t, the final noise representation approaches a standard Gaussian distribution.

The denoising process follows the Markov chain form, and the *true* denoising process from time step *t* to *s* < *t* is also expressed in a closed form by considering the original data *x*:

6 $$q({z}_{s}| {z}_{t},x)={\mathcal{N}}({z}_{s};{\mu }_{t\to s}(x,{z}_{t}),{\sigma }_{t\to s}^{2}{\bf{I}}),$$

where the mean *μ*_*t*→*s*_ and the variance ${\sigma }_{t\to s}^{2}$ are defined as follows:

7 $${\mu }_{t\to s}(x,{z}_{t})=\frac{{\alpha }_{t| s}{\sigma }_{s}^{2}}{{\sigma }_{t}^{2}}{z}_{t}+\frac{{\alpha }_{s}{\sigma }_{t| s}^{2}}{{\sigma }_{t}^{2}}x,\,\,{\rm{and}}\,\,{\sigma }_{t\to s}=\frac{{\sigma }_{t| s}{\sigma }_{s}}{{\sigma }_{t}},$$

with ${\alpha }_{t| s}=\frac{{\alpha }_{t}}{{\alpha }_{s}}$ and ${\sigma }_{t| s}={\sigma }_{t}^{2}-{\alpha }_{t| s}^{2}{\sigma }_{s}^{2}$, according to the notation of Hoogeboom et al.^43^. During the denoising process, the objective is to progressively remove noise and reconstruct the original data. However, the *true* denoising process includes the unknown original data *x* as its input; therefore, the process cannot be determined directly. Thus, generation becomes possible by replacing the original data *x* with the predicted values $\widehat{x}$ through the neural network, as follows:

8 $${p}_{\theta }({z}_{s}| {z}_{t})={\mathcal{N}}({z}_{s};{\mu }_{t\to s}(\widehat{x},{z}_{t}),{\sigma }_{t\to s}^{2}{\bf{I}}).$$

According to Ho et al.^42^, it is easier to predict the noise $\widehat{{\epsilon }_{\theta }}={\phi }_{\theta }({z}_{t},t)$ to be removed than to predict $\widehat{x}$ directly. Specifically, by rewriting Eq. (5) as *z*_*t*_ = *α*_*t*_*x* + *σ*_*t*_*ϵ* with $\epsilon \sim {\mathcal{N}}({\bf{0}},{\bf{I}})$ and replacing *ϵ* with $\widehat{\epsilon }$, $\widehat{x}$ can be expressed as shown below:

9 $$\widehat{x}=\frac{1}{{\alpha }_{t}}{z}_{t}-\frac{{\sigma }_{t}}{{\alpha }_{t}}\widehat{{\epsilon }_{\theta }}.$$

The learning objective of the generative model is to maximize the log-likelihood log*p*(*x*) of the observed data *x*. However, since this metrics is difficult to calculate directly, a variational lower bound (VLB)^43,62^ is introduced as follows:

10 $$-\log {p}_{\theta }(x)\le {L}_{T}+\mathop{\sum }\limits_{t=1}^{T}{L}_{t}+{L}_{0}.$$

*L*_*T*_ is the Kullback-Leibler (KL) divergence between the noise representation *q*(*z*_*T*_∣*x*) at time step *T* and the standard normal distribution $p({z}_{T})={\mathcal{N}}({\bf{0}},{\bf{I}})$ and is used to evaluate whether the diffusion process can be effectively approximated as a standard normal distribution. ${L}_{0}=-\log p(x| {z}_{0})$ is the reconstruction loss, which captures how well the model can reconstruct the original data *x* from the denoised latent variable *z*_0_. *L*_*t*_ is the loss term that enables the model to accurately predict the noise at each time step and is expressed as follows:

11 $${L}_{t}=-{D}_{KL}(q({z}_{t-1}| x,{z}_{t})\parallel {p}_{\theta }({z}_{t-1}| \widehat{x},{z}_{t}))$$

12 $$={{\mathbb{E}}}_{\epsilon \sim {\mathcal{N}}({\bf{0}},{\bf{I}})}\left[\frac{1}{2}\left(\frac{SNR(t-1)}{SNR(t)}-1\right)\parallel \epsilon -{\widehat{\epsilon }}_{\theta }({z}_{t},t){\parallel }^{2}\right],$$

where $SNR(t)={\alpha }_{t}^{2}/{\sigma }_{t}^{2}$ is the signal-to-noise ratio^62^. Minimizing the VLB improves the generative performance of the model. However, the denoising diffusion probabilistic model (DDPM)^42^ employs a simple loss function, i.e., the mean squared error between the true noise and the predicted noise, which is predicted from the noise representation *z*_*t*_. In practice, the loss function is minimized for a randomly sampled time step *t* ~ Uniform(0, *T*) as follows:

13 $${L}_{{\rm{train}}}=\frac{1}{2}\parallel \epsilon -{\widehat{\epsilon }}_{\theta }({z}_{t},t){\parallel }^{2}.$$

E(3)-equivariant graph convolutional layers (EGCLs)^36,43^ update both the coordinates *x* and node features *h*. The update expression ***x***^*l*+1^, ***h***^*l*+1^ = EGCL[***x***^*l*^, ***h***^*l*^] can be unfolded as follows:

14 $${{\boldsymbol{m}}}_{ij}={\phi }_{e}({{\boldsymbol{h}}}_{i}^{l},{{\boldsymbol{h}}}_{j}^{l},{d}_{ij}^{2},{a}_{ij})$$

15 $${\widetilde{e}}_{ij}={\phi }_{att}({{\boldsymbol{m}}}_{ij})$$

16 $${{\boldsymbol{h}}}_{i}^{l+1}={\phi }_{h}\left({{\boldsymbol{h}}}_{i}^{l},\mathop{\sum }\limits_{j\ne i}{\widetilde{e}}_{ij}{{\boldsymbol{m}}}_{ij}\right)$$

17 $${{\boldsymbol{r}}}_{i}^{l+1}=\left\{\begin{array}{ll}{{\boldsymbol{x}}}_{i}^{l}+\mathop{\sum }\limits_{j\ne i}\frac{{{\boldsymbol{x}}}_{i}^{l}-{{\boldsymbol{x}}}_{j}^{l}}{{d}_{ij}+1}{\phi }_{r}^{d}({{\boldsymbol{h}}}_{i}^{l},{{\boldsymbol{h}}}_{j}^{l},{d}_{ij}^{2},{a}_{ij}) & \\ +\frac{({{\boldsymbol{x}}}_{i}^{l}-{\bar{{\boldsymbol{x}}}}_{l})\times ({{\boldsymbol{x}}}_{j}^{l}-{\bar{{\boldsymbol{x}}}}_{l})}{\parallel ({{\boldsymbol{x}}}_{i}^{l}-{\bar{{\boldsymbol{x}}}}_{l})\times ({{\boldsymbol{x}}}_{j}^{l}-{\bar{{\boldsymbol{x}}}}_{l})\parallel +1}{\phi }_{x}^{\times }({{\boldsymbol{h}}}_{i}^{l},{{\boldsymbol{h}}}_{j}^{l},{d}_{ij}^{2},{a}_{ij}) & \,{\rm{if}}\,\,i\in \,{\rm{ligand}}\\ {{\boldsymbol{x}}}_{i}^{l} & \,{\rm{if}}\,\,i\in \,{\rm{protein\; or\; interaction}}\,,\end{array}\right.$$

where *ϕ*_*e*_, *ϕ*_*att*_, *ϕ*_*h*_ and *ϕ*_*r*_ are learnable multilayer perceptrons (MLPs), and ${d}_{ij}=\parallel {x}_{i}^{l}-{x}_{j}^{l}\parallel$ and *a*_*ij*_ are the Euclidean distances and edge features between nodes *i* and *j* respectively. Here, EGCL is equivariant with respect to the SE(3) group: EGCL($A{\mathcal{G}}$+*b*) = *A* EGCL(${\mathcal{G}}$) + *b*, where A is an orthogonal rotation matrix and b is a translation vector.

### Data construction

In this study, training and test datasets are conducted using the CrossDocked2020 dataset^10^, which contains approximately 22.5 million poses of ligands docked into multiple similar binding pockets across the entire Protein Data Bank. Following previously developed data preparation procedures^33,63^, binding poses with RMSDs greater than 1 Å are excluded. For a total of 184,057 complexes obtained, MMseq2^64^ is used to cluster the data according to the 30% sequence identity of the protein. From the resulting clusters, 100,000 protein-ligand pairs are randomly selected as the training set, and 100 pairs are randomly selected from the remaining clusters to construct the test set. In addition, interaction features are extracted from the protein-ligand complex data via the Open Drug Discovery Toolkit (ODDT)^65^ from the protein-ligand complex data. Hydrogen-bond interactions are identified based on donor-acceptor atom pairs within a distance cutoff of 3.5 Å, together with directional constraints defined by a base angle of 120^∘^ and a tolerance of ± 30^∘^. Hydrophobic interactions were defined as contacts between hydrophobic atom pairs within a distance cutoff of 4.0 Å.

### Training setting

DiffPharma was trained on 4 GPUs (H100) using the TSUBAME4.0 supercomputer at the Tokyo Institute of Science and the training time is approximately 120 hours. DiffPharma is trained from scratch, without using any pre-trained weights, following the training hyperparameters and architectural details summarized in Supplementary Table 1.

### Scope of applicability

Structure-based pharmacophore modeling extracts key interaction features from protein-ligand complexes. It is typically used as a filter to identify candidate molecules that satisfy interaction patterns, enabling virtual screening of existing compound libraries^17,66^. In contrast to interaction-based screening from predefined compound databases, DiffPharma enables the generation of molecules with specific interactions by directly incorporating this interaction information into the molecular generation process. This allows exploration of chemical space beyond existing databases. Therefore, DiffPharma is not intended for unconstrained de novo molecular discovery. It is designed as a scaffold hopping and interaction-guided lead optimization method, aiming to explore new molecules while preserving important binding interactions.

In standard DiffPharma usage, interaction patterns are extracted from experimentally determined or docking-derived complex structures, and treated as interaction constraints. Even when experimentally resolved ligand-bound complex structures are unavailable, or when the protein structure itself is unknown, DiffPharma can be applied within a practical structure-based drug design (SBDD) pipeline. Specifically, a three-dimensional structure of the target protein may first be obtained from its amino acid sequence using protein structure prediction methods. An existing compound library is evaluated against the predicted protein structure using docking methods to obtain candidate binding modes. Interaction patterns extracted from such predicted complexes are subsequently utilized as interaction constraints, enabling interaction-guided molecular generation even for target proteins lacking known ligands.

### Positioning in structure-based drug discovery

In SBDD, one traditional approach is database-driven virtual screening, which identifies candidate molecules by searching predefined compound libraries. More recently, machine learning-based end-to-end structure prediction frameworks, such as AlphaFold3^27^ and Boltz-2^28^, have been developed to predict the three-dimensional binding poses and binding affinities of existing ligands in complex with target proteins. In contrast, DiffPharma focuses on exploring new chemical structures given a target protein and specified interactions. Consequently, end-to-end structure prediction models and DiffPharama play complementary roles in structure-based drug discovery.

## Supplementary information

Supplementary information

## Data Availability

The training and test datasets, the generated molecules, and the pre-trained DiffPharma model used in this study are publicly available on Zenodo at 10.5281/zenodo.15428723.

## Code availability

The full implementation of the DiffPharma model, including the training and sampling code as well as the execution environment provided on Google Colab, is available under the MIT license from the GitHub repository: https://github.com/sekijima-lab/DiffPharma.
