# Supplementary material for: Interaction-constrained 3D molecular generation using a diffusion model enables structure-based pharmacophore modeling for drug design
Source: NPJ Drug Discov. 2026 Mar 2;3:8. doi: 10.1038/s44386-026-00040-x (PMC13267100; doi:10.1038/s44386-026-00040-x)
Supplement: Supplementary file 1 — Supplementary information [file 44386_2026_40_MOESM1_ESM.pdf]

Supplementary Information for: Interaction-constrained 3D Molecular Generation Using a Diffusion Model Enables Structure-based Pharmacophore Modeling for Drug Design

Masami Sako[1†], Nobuaki Yasuo[2†], Masakazu Sekijima[1*†]

[1*]Department of Computer Science, Institute of Science Tokyo, 4259–J3–23, Nagatsuta-cho, Midori-ku, Yokohama, 226-8501, Kanagawa, Japan.
[2]Department of Chemical Science and Engineering, Institute of Science Tokyo, S6-23, Ookayama, Meguro-ku, 152-8550, Tokyo, Japan.

*Corresponding author(s). E-mail(s): sekijima@comp.isct.ac.jp;
Contributing authors: sako.m.b47c@m.isct.ac.jp; yasuo.n.0a87@m.isct.ac.jp;
†These authors contributed equally to this work.

1 Proof of E(3)-Equivariance in MAP-EGNN

The Multi-Path Adaptive Fusion E(3)-equivariant graph neural networks (MAP-EGNN) consists of a Biased Fusion block and a Uniform Fusion block which are combinations of multiple EGCLs. The Biased Fusion block performs asymmetric fusion processing for each data series. For the protein and ligand series, the E(3)-equivariance of the EGCLs themselves is preserved[1] since the output is not averaged:

$$EGCL_P(A \cdot x_L^l + b, h_L^l, A \cdot x_P^l + b, h_P^l) = A \cdot EGCL_P(x_L^l, h_L^l, x_P^l, h_P^l) + b. \tag{1}$$

For the hydrogen bond and ligand series, the Biased Fusion operation is defined for the output with an averaging process as follows:

$$h_L^{l+1}, x_L^{l+1} = \frac{1}{2}\left[EGCL_H(x_L^l, h_L^l, x_H^l, h_H^l) + EGCL_P(x_L^l, h_L^l, x_P^l, h_P^l)\right]. \tag{2}$$

The Biased Fusion operation for this series preserves E(3)-equivariance as follows:

$$\frac{1}{2}\left[EGCL_H(A \cdot x_L^l + b, h_L^l, A \cdot x_H^l + b, h_H^l) + EGCL_P(A \cdot x_L^l + b, h_L^l, A \cdot x_P^l + b, h_P^l)\right] \tag{3}$$

$$= \frac{1}{2}\left[A \cdot EGCL_H(x_L^l, h_L^l, x_H^l, h_H^l) + b + A \cdot EGCL_P(x_L^l, h_L^l, x_P^l, h_P^l) + b\right] \tag{4}$$

$$= A \cdot \frac{1}{2}\left[EGCL_H(x_L^l, h_L^l, x_H^l, h_H^l) + EGCL_P(x_L^l, h_L^l, x_P^l, h_P^l)\right] + b. \tag{5}$$

Similarly, the hydrophobic interaction and ligand series also maintains E(3)-equivariance.

$$\frac{1}{2}\Big[EGCL_{HP}(A \cdot x_L^l + b, h_L^l, A \cdot x_{HP}^l + b, h_{HP}^l) \tag{6}$$

$$+EGCL_P(A \cdot x_L^l + b, h_L^l, A \cdot x_P^l + b, h_P^l)\Big] \tag{7}$$

$$= A \cdot \frac{1}{2}\Big[EGCL_{HP}(x_L^l, h_L^l, x_{HP}^l, h_{HP}^l) + EGCL_P(x_L^l, h_L^l, x_P^l, h_P^l)\Big] + b \tag{8}$$

From the above, it can be shown that the Biased Fusion block consisting of L layers BF^L satisfies E(3)-equivariance as follows:

$$\mathrm{BF}^L(A \cdot G + b) = A \cdot \mathrm{BF}^L(G) + b. \tag{9}$$

Unlike the Biased Fusion block, which applies interaction-specific processing, the Uniform Fusion block performs a symmetric fusion operation by equally averaging the outputs of all data series. This fusion process also satisfies E(3)-equivariance as follows:

$$\frac{1}{3}\sum_{t \in \{p,h,hp\}} EGCL_t(A \cdot x_L^l + b, h_L^l, A \cdot x_t^l + b, h_t^l) \tag{10}$$

$$= \frac{1}{3}\sum_{t \in \{P,H,HP\}} \left[A \cdot EGCL_t(x_L^l, h_L^l, x_t^l, h_t^l) + b\right] \tag{11}$$

$$= A \cdot \left(\frac{1}{3}\sum_{t \in \{P,H,HP\}} EGCL_t(x_L^l, h_L^l, x_t^l, h_t^l)\right) + b, \tag{12}$$

leading to the Uniform Fusion block consisting of M layers UF^M satisfying E(3)-equivariance as shown below:

$$\mathrm{UF}^M(A \cdot G + b) = A \cdot \mathrm{UF}^M(G) + b. \tag{13}$$

Therefore, it follows that the entire MAP-EGNN also satisfies E(3)-equivariance as follows:

$$\text{MAP-EGNN}(A \cdot G + b) = A \cdot \text{MAP-EGNN}(G) + b. \tag{14}$$

2 Hyperparameters

Table 1: Hyperparameters for DiffPharma.

Hyperparameters	Values
Number of diffusion steps	500
Batch size	32
Number of Epoch	1000
Number of Epoch	1.0×10^{-3}
Joint embedding dim.	128
Hidden dim.	256
Edge cutoff distance for ligand-ligand interactions	fully connected
Edge cutoff distance for others[1]	5 Å
Number of Biased Fusion block layers: L	3
Number of Uniform Fusion block layers: M	1
Number of MAP-EGNN layers: N	2

[1] ligand-protein, protein-protein, ligand-hydrogen, hydrogen-hydrogen, ligand-hydrophobic, and hydrophobic-hydrophobic interactions

3 Interaction Count Statistics of the Training and Test Sets

The number of protein–ligand interactions observed in the CrossDocked-derived training and test sets is summarized. Interaction patterns are identified for protein–ligand pairs using the Open Drug Discovery Toolkit (ODDT)[2] in an atom-by-atom manner. Supplementary Table 2 reports the average number of interactions for each interaction type, while Supplementary Fig. 1 presents the distributions of hydrogen-bond and hydrophobic-interaction patterns per protein–ligand pair in the training and test sets.

Hydrogen bonds and hydrophobic interactions are prevalent across both datasets. Specifically, the fraction of protein–ligand pairs that contain at least one interaction is 94% (training) and (92%) (test) for hydrogen bonds, and 84% (training) and 76% (test) for hydrophobic interactions. In contrast, the CrossDocked dataset contains relatively few examples of interaction types such as π–π stacking, cation–π interactions, salt bridges, and halogen bonds.

Table 2: Average number of protein–ligand interaction events in the training and test sets.

Interaction type	Training	Test
Hydrogen bond	5.9	5.4
Hydrophobic interaction	10.6	8.7
π–π stacking	0.05	0.04
Cation–π	0.03	0.02
Salt bridge	0.01	0.01
Halogen bond	0.01	0.00

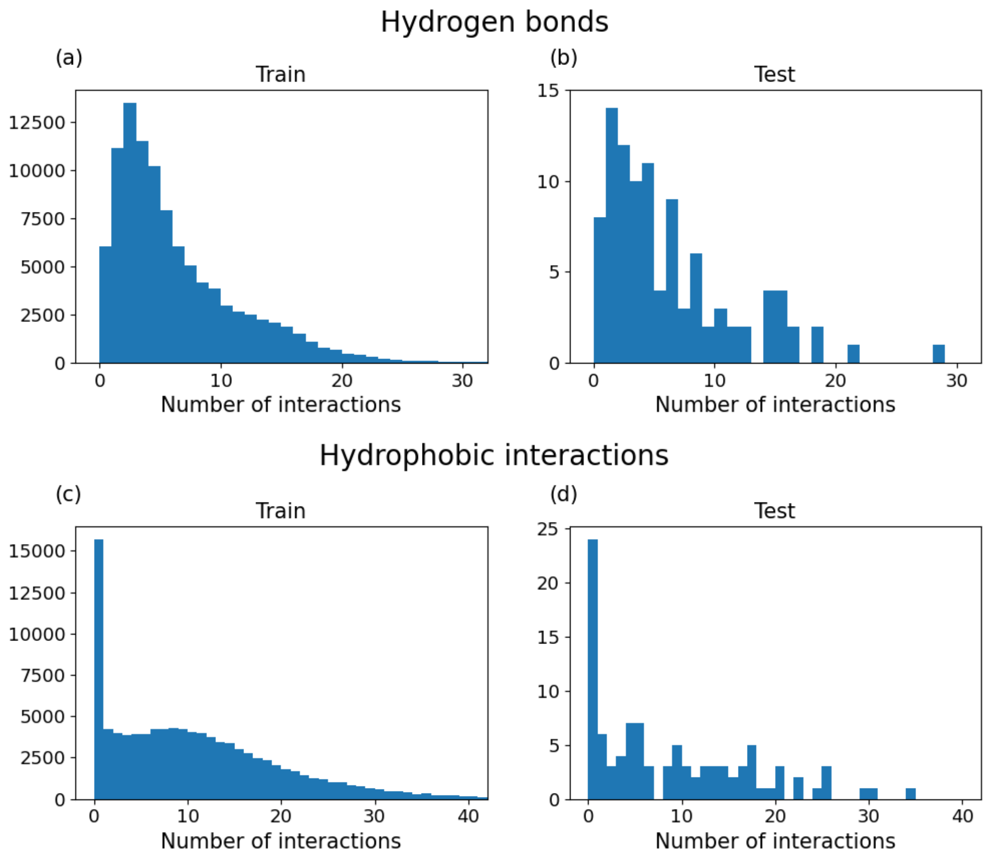

Supplementary Fig. 1: The number of interaction per protein–ligand pair for hydrogen bonds and hydrophobic interactions identified using the Open Drug Discovery Toolkit (ODDT) in an atom-by-atom manner.(a,b) correspond to hydrogen-bond interaction counts in the training and test sets, and (c, d) correspond to hydrophobic-interaction counts in the training and test sets. Figures generated using Matplotlib[3].

4 Interaction Reproducibility Analyses

4.1 Summary Statistics of Interaction Reproducibility

Table 3: Mean and standard deviation of interaction reproducibility across targets. For each model, interaction reproducibility is quantified using cosine similarity and summarized as mean ± standard deviation across 100 target proteins. Hydrogen-bond, hydrophobic, and total interaction similarities correspond to the target-wise averages computed from 100 generated molecules per target.

Model	hydrogen bond	hydrophobic interaction	Total
Pocket2Mol	0.41 ± 0.20	0.52 ± 0.20	0.49 ± 0.15
FLAG	0.46 ± 0.24	0.51 ± 0.20	0.49 ± 0.19
DiffSBDD	0.47 ± 0.17	0.51 ± 0.15	0.49 ± 0.12
DeepICL	0.41 ± 0.20	0.64 ± 0.15	0.54 ± 0.14
MolCRAFT	0.59 ± 0.21	0.55 ± 0.19	0.59 ± 0.15
DiffInt	0.80 ± 0.15	0.45 ± 0.16	0.62 ± 0.14
DiffPharma	0.80 ± 0.13	0.83 ± 0.14	0.83 ± 0.09

4.2 Interaction reproducibility across targets

Table 4: Cosine similarity scores of residue-wise hydrogen-bond interaction patterns between each generated molecule and its corresponding reference ligand. The values are averaged over 100 generated molecules for each of 100 target proteins. Results are shown separately for hydrogen bond, hydrophobic interaction, and total interaction patterns, across seven models (Pocket2Mol, FLAG, DiffSBDD, DeepICL, MolCRAFT, DiffInt, and DiffPharma).

	Hydrogen-bond interaction cosine similarity						
PDB	Pocket2Mol	FLAG	DiffSBDD	DeepICL	MolCRAFT	DiffInt	DiffPharma
14gs	0.22	0.72	0.32	0.18	0.46	0.67	0.87
1a2g	0.51	0.14	0.40	0.36	0.39	0.75	0.54
1afs	0.38	0.75	0.56	0.40	0.74	0.85	0.86
1ai4	0.53	0.00	0.49	0.70	0.80	0.80	0.80
1coy	-	-	-	-	-	-	-
1d7j	0.35	0.31	0.55	0.54	0.54	0.84	0.71
1djy	0.56	0.54	0.63	0.48	0.79	0.86	0.90
1dxo	0.08	0.11	0.03	0.32	0.07	0.74	0.83
1e8h	0.57	0.38	0.60	0.61	0.82	0.84	0.86
1fmc	0.21	0.49	0.32	0.15	0.50	0.91	0.77
1gg5	0.48	0.50	0.41	0.36	0.51	0.85	0.68
1h0i	0.36	0.18	0.55	0.49	0.48	0.78	0.71
1h36	-	-	-	-	-	-	-
1jn2	0.80	0.92	0.83	0.51	0.91	0.91	0.95
1k9t	-	-	-	-	-	-	-
1l3l	0.07	0.17	0.45	0.29	0.39	0.78	0.94
1phk	0.41	0.33	0.52	0.32	0.62	0.83	0.80
1r1h	0.47	0.44	0.59	0.67	0.71	0.81	0.44
1rs9	0.48	0.48	0.63	0.81	0.78	0.87	0.93
1umd	0.23	0.85	0.53	0.28	0.67	0.84	0.74
2azy	0.01	0.23	0.04	0.15	0.05	0.77	0.74
2cy0	0.66	0.36	0.68	0.58	0.75	0.95	0.93
2e24	0.22	0.61	0.44	0.30	0.45	0.83	0.83
2e6d	0.85	0.50	0.73	0.68	0.85	0.94	0.91
2f2c	0.50	0.65	0.49	0.43	0.60	0.90	0.91
2gns	0.19	0.00	0.21	0.05	0.10	0.23	0.93
2hcj	0.57	0.47	0.74	0.60	0.94	0.87	0.87
2jjg	0.66	0.66	0.72	0.65	0.79	0.87	0.85
2pc8	0.64	0.71	0.59	0.40	0.62	0.90	0.89
2pqw	0.11	0.56	0.24	0.28	0.43	0.81	0.81
2rhy	0.17	0.72	0.19	0.14	0.49	0.77	0.64
2rma	0.31	0.09	0.31	0.47	0.35	0.75	0.79
2v3r	0.27	0.43	0.42	0.36	0.44	0.75	0.63
2z3h	0.49	0.41	0.44	0.38	0.63	0.81	0.84
2zen	0.20	0.17	0.36	0.22	0.37	0.64	0.47
3af2	0.68	0.73	0.70	0.44	0.91	0.90	0.86
3b6h	-	-	-	-	-	-	-
3chc	0.58	0.82	0.62	0.71	0.80	0.95	0.93
3daf	0.41	0.51	0.55	0.30	0.62	0.72	0.75
3dzh	0.39	0.21	0.44	0.48	0.62	0.76	0.71
3ej8	0.78	0.67	0.44	0.88	0.77	0.91	0.89
3g51	0.60	0.46	0.56	0.53	0.75	0.76	0.77
3gs6	0.69	0.93	0.78	0.40	0.88	0.97	0.95
3hy9	0.50	0.83	0.56	0.58	0.69	0.86	0.79
3jyh	0.36	0.19	0.42	0.37	0.50	0.85	0.83
3kc1	0.62	0.10	0.55	0.45	0.72	0.84	0.85
3l3n	0.58	0.82	0.71	0.68	0.83	0.90	0.92
3li4	0.41	0.28	0.28	0.20	0.50	0.41	0.43

	Hydrogen-bond interaction cosine similarity (continued)						
PDB	Pocket2Mol	FLAG	DiffSBDD	DeepICL	MolCRAFT	DiffInt	DiffPharma
3nfb	0.61	0.43	0.65	0.65	0.77	0.91	0.86
3o96	0.12	0.12	0.15	0.14	0.09	0.50	0.63
3pdh	0.20	0.69	0.41	0.37	0.52	0.84	0.83
3pnm	0.54	0.47	0.47	0.38	0.52	0.83	0.71
3tym	0.45	0.48	0.24	0.25	0.58	0.78	0.84
3u5y	0.45	0.67	0.56	0.22	0.67	0.86	0.80
3u9f	0.19	0.21	0.33	0.02	0.31	0.82	0.73
3v4t	0.70	0.28	0.66	0.49	0.71	0.84	0.84
3w83	0.68	0.80	0.53	0.34	0.65	0.88	0.83
4aaw	0.71	0.50	0.67	0.55	0.80	0.94	0.93
4aua	0.63	0.65	0.45	0.64	0.58	0.75	0.57
4azf	0.17	0.49	0.35	0.51	0.33	0.76	0.80
4bel	0.61	0.62	0.62	0.49	0.65	0.95	0.73
4d7o	0.32	0.47	0.27	0.10	0.66	0.88	0.73
4f1m	0.42	0.55	0.41	0.27	0.65	0.79	0.85
4g3d	0.35	0.53	0.37	0.14	0.61	0.88	0.90
4gvd	-	-	-	-	-	-	-
4h3c	0.00	0.11	0.03	0.33	0.21	0.61	0.72
4iiy	0.23	0.45	0.57	0.45	0.78	0.90	0.86
4iwq	0.37	0.43	0.41	0.56	0.43	0.88	0.89
4ja8	0.36	0.12	0.60	0.92	0.74	0.94	0.96
4kcq	0.48	0.23	0.20	0.03	0.49	0.76	0.90
4keu	0.43	0.13	0.51	0.47	0.53	0.84	0.79
4lfu	0.14	0.77	0.53	0.43	0.46	0.83	0.90
4m7t	0.50	0.53	0.54	0.32	0.63	0.84	0.79
4p6p	0.74	0.73	0.80	0.72	0.94	0.89	0.87
4pxz	0.39	0.55	0.49	0.55	0.79	0.85	0.87
4q8b	-	-	-	-	-	-	-
4qlk	0.14	0.61	0.56	0.47	0.65	0.78	0.75
4rlu	0.29	0.19	0.45	0.28	0.42	0.82	0.86
4rn0	0.01	0.30	0.00	0.01	0.01	0.06	0.27
4rv4	0.63	0.54	0.60	0.56	0.91	0.79	0.85
4tos	0.09	0.18	0.34	0.61	0.33	0.78	0.61
4tqr	0.57	0.35	0.48	0.52	0.66	0.86	0.87
4u5s	0.46	0.10	0.47	0.28	0.51	0.68	0.69
4xli	0.63	0.74	0.43	0.77	0.57	0.85	0.83
4yhj	0.48	0.38	0.40	0.22	0.51	0.87	0.86
4z2g	0.46	0.30	0.45	0.13	0.51	0.87	0.63
4zfa	0.30	0.05	0.30	0.04	0.17	0.38	0.90
5aeh	0.11	0.48	0.45	0.36	0.45	0.84	0.69
5b08	0.46	0.80	0.59	0.63	0.78	0.92	0.98
5bur	0.34	0.52	0.39	0.34	0.76	0.86	0.92
5d7n	0.38	0.64	0.50	0.42	0.62	0.79	0.86
5i0b	0.42	0.29	0.36	0.40	0.48	0.36	0.73
5l1v	0.42	0.89	0.65	0.50	0.82	0.94	0.88
5liu	0.11	0.55	0.39	0.24	0.86	0.81	0.89
5mgl	0.54	0.59	0.44	0.67	0.69	0.81	0.84
5mma	-	-	-	-	-	-	-
5ngz	-	-	-	-	-	-	-
5q0k	0.04	0.13	0.10	0.00	0.20	0.78	0.81
5tjn	0.51	0.83	0.53	0.53	0.58	0.87	0.92
5w2g	0.51	0.47	0.58	0.48	0.61	0.79	0.85

Table 5: Cosine similarity scores of residue-wise hydrophobic interaction patterns between each generated molecule and its corresponding reference ligand. The values are averaged over 100 generated molecules for each of 100 target proteins. Results are shown separately for hydrogen bond, hydrophobic interaction, and total interaction patterns, across seven models (Pocket2Mol, FLAG, DiffSBDD, DeepICL, MolCRAFT, DiffInt, and DiffPharma).

	Hydrophobic interaction cosine similarity						
PDB	Pocket2Mol	FLAG	DiffSBDD	DeepICL	MolCRAFT	DiffInt	DiffPharma
14gs	0.65	0.62	0.46	0.63	0.69	0.53	0.91
1a2g	-	-	-	-	-	-	-
1afs	0.62	0.56	0.41	0.64	0.60	0.33	0.88
1ai4	0.93	0.00	0.65	0.84	0.80	0.62	0.96
1coy	0.51	0.49	0.43	0.59	0.48	0.41	0.90
1d7j	0.54	0.58	0.63	0.60	0.69	0.22	0.72
1djy	-	-	-	-	-	-	-
1dxo	0.58	0.52	0.44	0.80	0.63	0.36	0.88
1e8h	-	-	-	-	-	-	-
1fmc	0.60	0.71	0.44	0.53	0.48	0.19	0.87
1gg5	0.50	0.64	0.51	0.69	0.54	0.49	0.93
1h0i	0.32	0.36	0.38	0.26	0.22	0.51	0.82
1h36	0.67	0.61	0.66	0.63	0.70	0.46	0.82
1jn2	0.10	0.22	0.16	0.59	0.23	0.13	0.69
1k9t	-	-	-	-	-	-	-
1l3l	0.70	0.55	0.64	0.70	0.67	0.52	0.93
1phk	-	-	-	-	-	-	-
1r1h	0.78	0.70	0.73	0.66	0.78	0.60	0.84
1rs9	0.38	0.61	0.54	0.87	0.50	0.50	0.89
1umd	0.49	0.83	0.69	0.77	0.74	0.55	0.84
2azy	0.75	0.71	0.69	0.71	0.80	0.65	0.94
2cy0	0.43	0.42	0.45	0.53	0.14	0.40	0.81
2e24	0.68	0.38	0.68	0.74	0.49	0.51	0.90
2e6d	-	-	-	-	-	-	-
2f2c	0.76	0.66	0.60	0.50	0.67	0.59	0.92
2gns	0.75	0.63	0.52	0.76	0.55	0.72	0.99
2hcj	-	-	-	-	-	-	-
2jjg	0.48	0.49	0.59	0.85	0.59	0.61	0.89
2pc8	0.31	0.44	0.48	0.64	0.44	0.51	0.92
2pqw	0.56	0.74	0.56	0.62	0.59	0.53	0.83
2rhy	0.48	0.68	0.47	0.70	0.51	0.38	0.75
2rma	0.38	0.15	0.45	0.53	0.67	0.39	0.88
2v3r	0.78	0.57	0.63	0.71	0.65	0.53	0.96
2z3h	0.68	0.54	0.66	0.66	0.71	0.59	0.89
2zen	0.21	0.69	0.55	0.74	0.41	0.35	0.94
3af2	-	-	-	-	-	-	-
3b6h	0.48	0.48	0.40	0.62	0.47	0.32	0.83
3chc	-	-	-	-	-	-	-
3daf	0.30	0.35	0.21	0.52	0.40	0.23	0.57
3dzh	0.01	0.01	0.08	0.17	0.03	0.11	0.80
3ej8	0.15	0.12	0.30	0.48	0.07	0.18	0.72
3g51	-	-	-	-	-	-	-
3gs6	0.00	0.40	0.00	0.13	0.00	0.00	0.09
3hy9	0.35	0.77	0.43	0.67	0.57	0.35	0.77
3jyh	0.70	0.67	0.58	0.72	0.71	0.64	0.82
3kc1	0.66	0.51	0.59	0.84	0.69	0.73	0.91
3l3n	0.42	0.49	0.39	0.62	0.37	0.43	0.73
3li4	0.47	0.30	0.61	0.76	0.69	0.38	0.92
3nfb	0.69	0.26	0.70	0.75	0.83	0.63	0.90
3o96	0.67	0.17	0.59	0.62	0.70	0.54	0.87
3pdh	0.59	0.41	0.53	0.67	0.59	0.55	0.87

PDB	Hydrophobic interaction cosine similarity (continued)						
	Pocket2Mol	FLAG	DiffSBDD	DeepICL	MolCRAFT	DiffInt	DiffPharma
3pnm	-	-	-	-	-	-	-
3tym	0.61	0.51	0.57	0.59	0.69	0.58	0.87
3u5y	0.30	0.52	0.41	0.53	0.42	0.14	0.59
3u9f	0.26	0.38	0.29	0.46	0.37	0.40	0.83
3v4t	-	-	-	-	-	-	-
3w83	-	-	-	-	-	-	-
4aaw	0.52	0.55	0.53	0.72	0.64	0.43	0.77
4aua	0.43	0.54	0.52	0.55	0.45	0.47	0.86
4azf	0.65	0.49	0.52	0.71	0.60	0.51	0.95
4bel	0.53	0.80	0.68	0.77	0.64	0.46	0.71
4d7o	0.60	0.45	0.54	0.56	0.73	0.46	0.89
4f1m	-	-	-	-	-	-	-
4g3d	0.58	0.80	0.62	0.66	0.70	0.62	0.98
4gvd	0.62	0.66	0.57	0.74	0.67	0.85	0.98
4h3c	0.61	0.63	0.61	0.74	0.77	0.63	0.93
4iiy	-	-	-	-	-	-	-
4iwq	0.64	0.51	0.61	0.62	0.69	0.45	0.94
4ja8	0.61	0.50	0.69	0.76	0.75	0.56	0.78
4kcq	0.59	0.61	0.51	0.74	0.65	0.40	0.94
4keu	-	-	-	-	-	-	-
4lfu	0.51	0.69	0.50	0.76	0.55	0.44	0.91
4m7t	0.10	0.23	0.09	0.18	0.11	0.10	0.48
4p6p	-	-	-	-	-	-	-
4pxz	-	-	-	-	-	-	-
4q8b	0.42	0.66	0.38	0.72	0.48	0.38	0.80
4qlk	-	-	-	-	-	-	-
4rlu	0.70	0.09	0.50	0.78	0.57	0.46	0.92
4rn0	0.41	0.70	0.52	0.55	0.54	0.37	0.82
4rv4	-	-	-	-	-	-	-
4tos	0.57	0.51	0.61	0.61	0.68	0.41	0.71
4tqr	-	-	-	-	-	-	-
4u5s	0.89	0.69	0.63	0.96	0.47	0.65	0.96
4xli	0.43	0.77	0.56	0.50	0.59	0.49	0.88
4yhj	-	-	-	-	-	-	-
4z2g	0.44	0.54	0.45	0.67	0.51	0.49	0.38
4zfa	0.72	0.49	0.65	0.75	0.74	0.63	0.94
5aeh	0.61	0.45	0.49	0.70	0.55	0.43	0.83
5b08	0.76	0.78	0.69	0.77	0.75	0.51	0.95
5bur	-	-	-	-	-	-	-
5d7n	0.36	0.65	0.40	0.54	0.40	0.36	0.81
5i0b	0.54	0.72	0.63	0.69	0.62	0.68	0.95
5l1v	0.60	0.60	0.46	0.48	0.59	0.41	0.78
5liu	0.79	0.84	0.73	0.82	0.77	0.57	0.93
5mgl	0.32	0.12	0.25	0.66	0.30	0.12	0.70
5mma	0.31	0.29	0.36	0.54	0.33	0.24	0.55
5ngz	0.11	0.01	0.41	0.57	0.29	0.27	0.82
5q0k	0.52	0.58	0.58	0.60	0.61	0.47	0.83
5tjn	0.49	0.32	0.47	0.42	0.34	0.55	0.76
5w2g	-	-	-	-	-	-	-

Table 6: Cosine similarity scores of residue-wise total interaction patterns, combining hydrogen-bond and hydrophobic interactions, between each generated molecule and its corresponding reference ligand. The values are averaged over 100 generated molecules for each of 100 target proteins. Results are shown separately for hydrogen bond, hydrophobic interaction, and total interaction patterns, across seven models (Pocket2Mol, FLAG, DiffSBDD, DeepICL, MolCRAFT, DiffInt, and DiffPharma).

	Total interaction cosine similarity						
PDB	Pocket2Mol	FLAG	DiffSBDD	DeepICL	MolCRAFT	DiffInt	DiffPharma
14gs	0.52	0.66	0.41	0.53	0.62	0.58	0.90
1a2g	0.37	0.11	0.30	0.30	0.25	0.43	0.51
1afs	0.58	0.63	0.47	0.60	0.66	0.45	0.87
1ai4	0.78	0.00	0.58	0.79	0.80	0.66	0.88
1coy	0.47	0.43	0.36	0.56	0.41	0.39	0.86
1d7j	0.50	0.51	0.61	0.58	0.64	0.49	0.73
1djy	0.49	0.47	0.58	0.43	0.76	0.76	0.89
1dxo	0.46	0.41	0.33	0.71	0.48	0.46	0.87
1e8h	0.50	0.29	0.50	0.52	0.78	0.82	0.86
1fmc	0.44	0.60	0.39	0.39	0.49	0.55	0.81
1gg5	0.50	0.60	0.48	0.67	0.53	0.55	0.89
1h0i	0.35	0.30	0.48	0.38	0.36	0.60	0.78
1h36	0.62	0.57	0.60	0.63	0.63	0.45	0.81
1jn2	0.67	0.83	0.71	0.54	0.84	0.69	0.92
1k9t	-	-	-	-	-	-	-
1l3l	0.62	0.49	0.61	0.65	0.63	0.59	0.93
1phk	0.23	0.18	0.36	0.22	0.51	0.80	0.79
1r1h	0.65	0.60	0.67	0.68	0.74	0.75	0.74
1rs9	0.47	0.55	0.60	0.85	0.67	0.65	0.91
1umd	0.37	0.84	0.59	0.54	0.70	0.75	0.79
2azy	0.65	0.64	0.60	0.64	0.69	0.70	0.92
2cy0	0.63	0.36	0.65	0.57	0.68	0.81	0.92
2e24	0.41	0.59	0.52	0.47	0.47	0.74	0.85
2e6d	0.83	0.46	0.70	0.67	0.84	0.82	0.87
2f2c	0.59	0.65	0.51	0.45	0.61	0.78	0.91
2gns	0.66	0.51	0.49	0.64	0.49	0.63	0.98
2hcj	0.54	0.46	0.65	0.57	0.89	0.86	0.87
2jjg	0.64	0.62	0.67	0.70	0.76	0.78	0.86
2pc8	0.54	0.66	0.55	0.51	0.58	0.72	0.90
2pqw	0.32	0.61	0.35	0.41	0.48	0.65	0.80
2rhy	0.39	0.72	0.36	0.53	0.52	0.47	0.70
2rma	0.37	0.14	0.39	0.51	0.51	0.56	0.83
2v3r	0.57	0.48	0.51	0.60	0.54	0.60	0.85
2z3h	0.55	0.44	0.50	0.47	0.65	0.75	0.85
2zen	0.22	0.57	0.45	0.61	0.36	0.40	0.82
3af2	0.62	0.70	0.65	0.39	0.85	0.87	0.85
3b6h	0.45	0.39	0.34	0.57	0.41	0.30	0.76
3chc	0.44	0.76	0.42	0.56	0.60	0.59	0.82
3daf	0.35	0.45	0.43	0.39	0.52	0.59	0.73
3dzh	0.25	0.14	0.33	0.37	0.44	0.56	0.74
3ej8	0.61	0.55	0.38	0.65	0.59	0.57	0.81
3g51	0.41	0.24	0.38	0.45	0.54	0.68	0.75
3gs6	0.52	0.81	0.57	0.30	0.63	0.73	0.81
3hy9	0.44	0.80	0.51	0.63	0.63	0.64	0.79
3jyh	0.57	0.52	0.53	0.61	0.63	0.72	0.85
3kc1	0.63	0.25	0.57	0.59	0.70	0.80	0.88
3l3n	0.58	0.79	0.65	0.66	0.78	0.77	0.88
3li4	0.43	0.30	0.50	0.66	0.60	0.38	0.81
3nfb	0.66	0.39	0.68	0.71	0.80	0.79	0.89
3o96	0.53	0.15	0.46	0.54	0.55	0.54	0.83
3pdh	0.49	0.50	0.50	0.61	0.57	0.64	0.86

	Total interaction cosine similarity (continued)						
PDB	Pocket2Mol	FLAG	DiffSBDD	DeepICL	MolCRAFT	DiffInt	DiffPharma
3pnm	0.51	0.40	0.37	0.31	0.46	0.58	0.67
3tym	0.58	0.50	0.48	0.50	0.65	0.67	0.87
3u5y	0.38	0.61	0.48	0.44	0.55	0.53	0.77
3u9f	0.26	0.31	0.30	0.36	0.36	0.50	0.79
3v4t	0.60	0.22	0.57	0.41	0.65	0.78	0.83
3w83	0.68	0.80	0.51	0.29	0.65	0.73	0.80
4aaw	0.61	0.54	0.59	0.66	0.70	0.69	0.86
4aua	0.50	0.56	0.51	0.57	0.51	0.55	0.80
4azf	0.49	0.48	0.45	0.64	0.50	0.60	0.88
4bel	0.57	0.74	0.66	0.68	0.64	0.73	0.75
4d7o	0.54	0.46	0.47	0.49	0.72	0.60	0.87
4f1m	0.26	0.32	0.23	0.21	0.47	0.59	0.82
4g3d	0.49	0.69	0.52	0.51	0.66	0.75	0.95
4gvd	0.48	0.58	0.40	0.73	0.46	0.76	0.92
4h3c	0.57	0.57	0.55	0.71	0.73	0.63	0.89
4iiy	0.17	0.36	0.47	0.36	0.72	0.87	0.85
4iwq	0.58	0.50	0.56	0.62	0.60	0.63	0.92
4ja8	0.59	0.45	0.68	0.77	0.75	0.61	0.80
4kcq	0.54	0.48	0.40	0.63	0.57	0.49	0.93
4keu	0.25	0.07	0.33	0.35	0.34	0.53	0.73
4lfu	0.45	0.72	0.51	0.70	0.53	0.54	0.91
4m7t	0.36	0.43	0.42	0.27	0.51	0.76	0.77
4p6p	0.63	0.59	0.70	0.68	0.90	0.73	0.85
4pxz	0.27	0.44	0.36	0.46	0.66	0.81	0.85
4q8b	0.40	0.65	0.32	0.70	0.46	0.36	0.74
4qlk	0.09	0.46	0.42	0.39	0.48	0.72	0.74
4rlu	0.56	0.15	0.48	0.61	0.52	0.62	0.88
4rn0	0.21	0.50	0.23	0.35	0.25	0.24	0.54
4rv4	0.62	0.50	0.57	0.53	0.90	0.78	0.85
4tos	0.47	0.44	0.53	0.63	0.61	0.48	0.70
4tqr	0.56	0.35	0.42	0.47	0.64	0.76	0.85
4u5s	0.65	0.45	0.57	0.70	0.50	0.64	0.84
4xli	0.50	0.76	0.53	0.58	0.59	0.59	0.87
4yhj	0.30	0.25	0.25	0.17	0.40	0.64	0.84
4z2g	0.45	0.48	0.45	0.58	0.51	0.63	0.57
4zfa	0.70	0.45	0.62	0.71	0.70	0.62	0.93
5aeh	0.53	0.47	0.48	0.66	0.54	0.54	0.81
5b08	0.70	0.79	0.67	0.74	0.76	0.67	0.96
5bur	0.26	0.43	0.31	0.26	0.71	0.76	0.90
5d7n	0.35	0.64	0.44	0.48	0.54	0.57	0.83
5i0b	0.52	0.61	0.55	0.62	0.57	0.62	0.91
5l1v	0.58	0.76	0.56	0.51	0.73	0.67	0.85
5liu	0.75	0.82	0.69	0.79	0.78	0.60	0.93
5mgl	0.43	0.36	0.34	0.66	0.47	0.40	0.77
5mma	0.25	0.26	0.22	0.47	0.20	0.18	0.40
5ngz	0.09	0.00	0.33	0.51	0.22	0.26	0.66
5q0k	0.47	0.54	0.52	0.55	0.56	0.52	0.84
5tjn	0.51	0.73	0.52	0.50	0.53	0.70	0.89
5w2g	0.33	0.33	0.39	0.36	0.42	0.65	0.82

4.3 Ablation Study

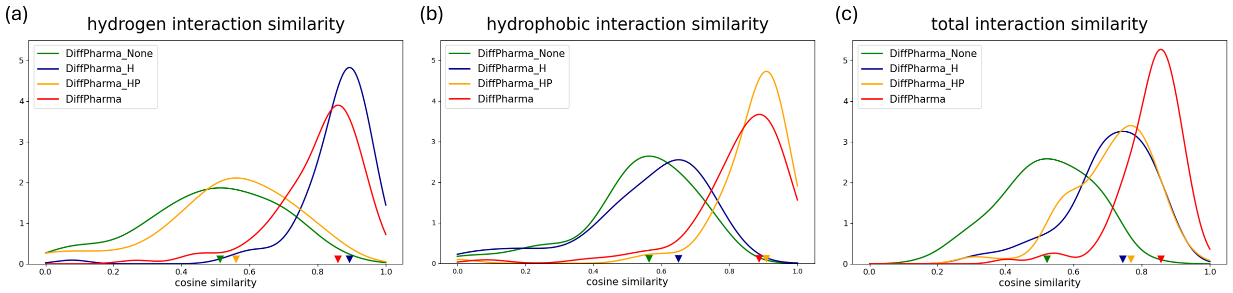

Supplementary Fig. 2: Cosine similarity of the residue-by-residue interaction patterns for **a** hydrogen bonds, **b** hydrophobic interactions, and **c** total interactions. The metrics are evaluated for DiffPharma and three ablation models. For each of the 100 target proteins contained in the test dataset, 100 molecules are generated per model, and the average similarity to the reference ligand is plotted. The downward triangles indicate the peak positions of the similarity distributions for the different models. Figures generated using Matplotlib.

DiffPharma consists of three distinct modules: a protein module, a hydrogen bond interaction module, and a hydrophobic interaction module. Among these modules, the latter two play crucial roles in reproducing protein–ligand interactions. To assess the individual contributions of the interaction modules, three types of ablation studies are conducted, with a focus on the impacts of the models on each type of interaction reproducibility. Specifically, three ablation models are evaluated: DiffPharma-None includes no interaction modules, DiffPharma-H retains only the hydrogen bond module, and DiffPharma-HP retains only the hydrophobic module. Each model is trained using the same hyperparameters as those of DiffPharma and evaluated on the test set using an identical validation protocol as shown in Supplementary Fig. 2.

The base model without any interaction modules, DiffPharma-None, yields no significant reproducibility difference between hydrogen bonds and hydrophobic interactions, with both distributions peaking at approximately 0.5. The hydrogen bond specific model, DiffPharma-H, achieves slightly better reproducibility for hydrogen bonds than DiffPharma does, while its performance in terms of hydrophobic interactions remains comparable to those of the base model. Conversely, the hydrophobic specific model, DiffPharma-HP, exhibits slightly improved reproducibility for hydrophobic interactions relative to DiffPharma, but exhibits similar performance to the base model for hydrogen bonds.

Notably, DiffPharma maintains comparable performance to each of the specialized models interms of their respective interaction types. This result suggests that the dedicated modules for hydrogen bonds and hydrophobic interactions, combined with an effective semantic integration scheme implemented through the MAP-EGNN architecture, contribute to the overall balanced performance of the proposed approach.

4.4 π–π Stacking Interaction Analysis

Although DiffPharma does not explicitly model π–π stacking interactions as independent interaction constraints, π–π stacking constraints are treated as a subset of hydrophobic interactions, which are imposed only through a distance-based constraint during generation. Because no directional or angular requirements specific to π–π stacking are conditioned, this experiment assesses whether π–π stacking interactions can arise implicitly under the simplified hydrophobic distance constraint. π–π stacking interactions between the generated molecules and the protein are identified using the Open Drug Discovery Toolkit (ODDT)[2]. In ODDT, π–π stacking is defined based on aromatic ring pairs within a distance cutoff of 5.0 Å, together with angular criteria corresponding to parallel or perpendicular ring arrangements, with an angular tolerance of $\pm 30°$. Among the 100 protein–ligand complexes in the test dataset, three reference structures (PDB IDs: 3dzh, 4bel, and 4iiy) contain π–π stacking interactions. For each of these targets, 100 molecules were generated following the same protocol described in the main text (Section 2.2). Cosine similarities are calculated specifically for π–π stacking interaction patterns between generated molecules and reference ligands, yielding π–π stacking interaction reproducibility rates of 7 %, 29 %, and 9 % for 3dzh, 4bel, and 4iiy, respectively.

5 Property Analysis

5.1 Molecular Property Analysis

The molecular properties of the training data, the test data, and the molecules generated by each model are listed in Supplementary Table 7. In terms of molecular weights, MolCRAFT[4], DiffInt[5], and DiffPharma produce molecules whose average values are closest to the test set, whereas other models tend to generate molecules that are either larger (e.g., DiffSBDD[6]) or smaller (e.g., Pocket2Mol[7], FLAG[8], DeepICL[9]), suggesting that autoregressive models tend to generate smaller structures.

With respect to the quantitative estimate of drug-likeness (QED)[10] where higher values are generally preferred, DeepICL yields the highest scores among all models. In contrast, DiffPharma has a lower QED, but its value is closest to that of the test data. For other properties, such as logP [11] (the logarithm of the partition coefficient of the solvent between octanol and water), the numbers of hydrogen bond donors and acceptors, and Lipinski[12] (how many rules in Lipinski's rule of five molecules are satisfied), DiffPharma exhibits a higher degree of agreement with the test data. The compared models are all structure-based molecular generation methods that focus on reproducing the reference molecules based on target structure information rather than explicitly optimizing individual physicochemical properties. From this perspective, the consistency of the properties with the test data suggests that DiffPharma better captures the underlying molecular characteristics that are relevant to the given task. The synthetic accessibility (SA) scores[13] of the diffusion-based models tends to be lower, and DiffPharma follows this trend. Finally, although DiffPharma has slightly lower molecular diversity than DeepICL and DiffInt, it still maintains a high level of structural diversity. Diversity is computed within each target protein as the average pairwise dissimilarity among all generated molecules (1 - Tanimoto similarity) using RDKit fingerprint[14], and the reported value is averaged across proteins.

Table 7: Comparison among the properties of training data, the test data and the generated molecules.

	molWt	QED	logP	Donors	Acceptors	Lipinski	SA	Diversity
training	339	0.534	1.76	2.73	5.44	4.49	0.755	-
test	332	0.476	0.89	3.35	6.08	4.27	0.723	-
Pocke2Mol	248	0.566	1.65	1.70	3.74	4.87	0.748	0.736
FLAG	299	0.487	1.44	2.57	4.70	4.57	0.701	0.701
DiffSBDD	353	0.469	1.21	3.04	5.93	4.53	0.578	0.728
DeepICL	281	0.588	3.49	1.42	2.42	4.66	0.771	0.797
MolCRAFT	322	0.504	1.16	3.49	5.00	4.46	0.686	0.725
DiffInt	327	0.516	1.55	2.96	4.91	4.64	0.605	0.750
DiffPharma	320	0.472	0.82	3.16	5.61	4.51	0.625	0.737

Finally, the potential trade-off between interaction similarity and internal diversity is examined across targets as shown in Supplementary Fig 3. The relationship is quantified for each target using Spearman's rank correlation. A statistically significant negative correlation was observed ($\rho = -0.41, \quad p = 2.5 \times 10^{-5}$), indicating a moderate trade-off between constraint satisfaction and molecular diversity. Notably, the average diversity across targets remained high (mean = 0.74), suggesting that DiffPharma preserves substantial structural diversity even under strong interaction constraints.

5.2 Nearest-Neighbor Similarity Analysis

For each generated molecule (N = 10,000), the nearest-neighbor similarity to the training and test sets is quantified as the maximum Tanimoto similarity computed with RDKit fingerprints. The average nearest-neighbor similarity is 0.51 with respect to the training set and 0.42 with respect to the test set. Exact matches based on canonical SMILES are observed for 80 generated molecules (0.8%) against the training set and 11 molecules (0.1%) against the test set. These results indicate that exact reproduction of training or reference ligands is rare, and that DiffPharma does not rely on memorization.

5.3 Substructure Analysis

To evaluate the 3D stability of the generated molecules, bond lengths for six types (C-C sp3, C-C sp2, C-N, C-O, C=O, C-C aromatic), bond angles for seven types (CCC, CCO, CNC, OPO, NCC, CC=O, and COC) and dihedral angles for six types (CCCC, cccc, CCCO, OCCO, Cccc, and CC=CC) are analyzed using RDKit[14]. Supplementary Fig. 4, 5, and 6 shows the KL divergences between the bond length, bond

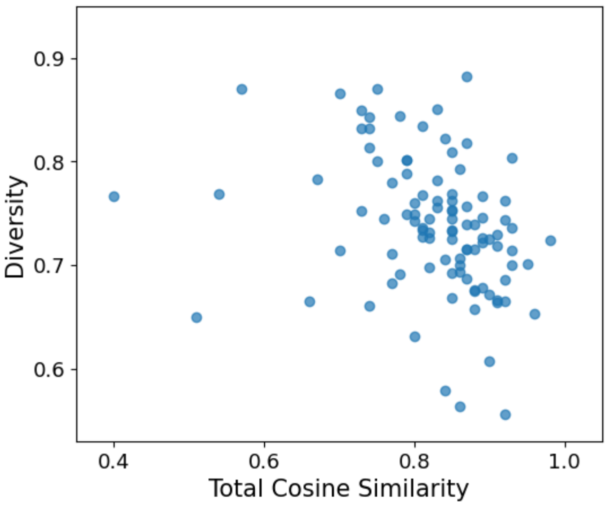

Supplementary Fig. 3: Relationship between interaction similarity and internal molecular diversity across targets. Figures generated using Matplotlib.

angle, and dihedral angle distributions of the test set and those of the molecules generated by each model, respectively. The corresponding KL divergence values are summarized in Supplementary Table 8.

For bond length distributions, all evaluated models show moderate KL divergences across the examined bond types, indicating no apparent breakdown of basic bond-length geometry in the generated molecules. In particular, for C–C aromatic bonds, DiffPharma exhibits relatively lower KL divergences compared with the other models, suggesting improved preservation of aromatic bond-length statistics. For bond angle distributions, DiffPharma shows a tendency toward higher KL divergences relative to other models, indicating lower reproducibility of certain local angle geometries. This trend is consistently observed across multiple angle types. For dihedral angle distributions, DiffPharma achieves KL divergences that are generally comparable to those of existing models, indicating effective preservation of global conformational characteristics. While some models achieve lower KL values for specific dihedral types, DiffPharma maintains competitive performance.

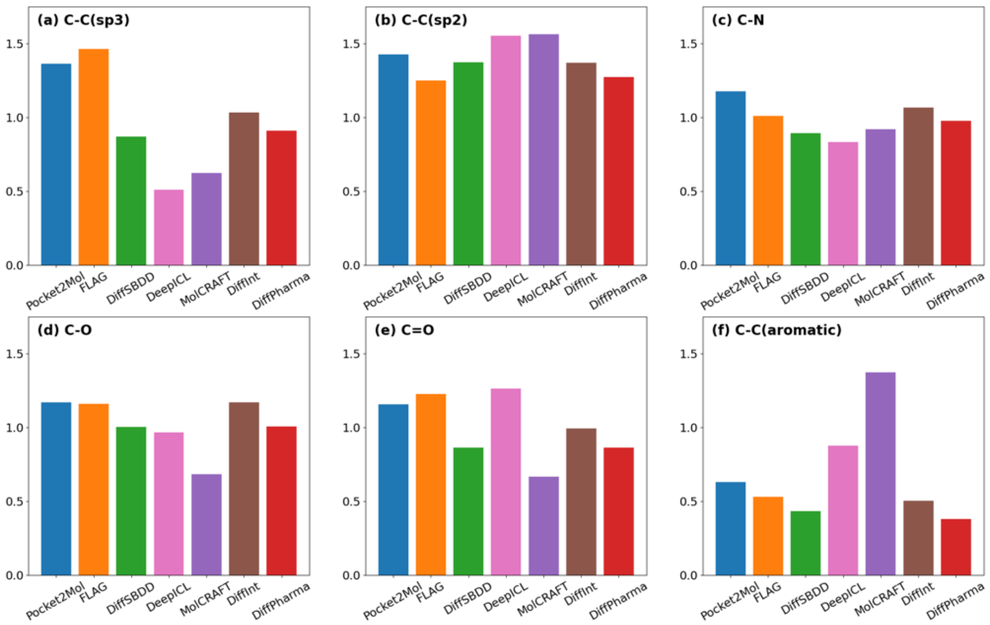

Supplementary Fig. 4: KL divergences between the bond length distributions of the test set and the molecules generated for each model. Figures generated using Matplotlib.

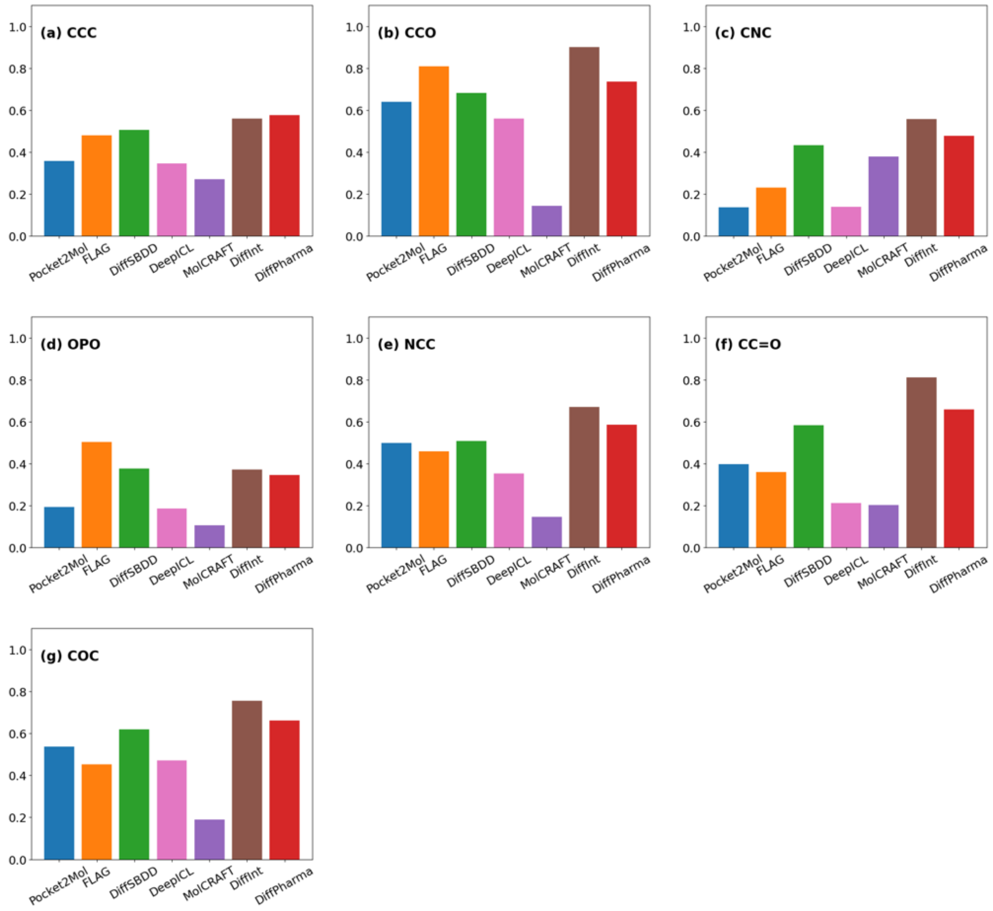

Supplementary Fig. 5: KL divergences between the bond angle distributions of the test set and the molecules generated for each model. Figures generated using Matplotlib.

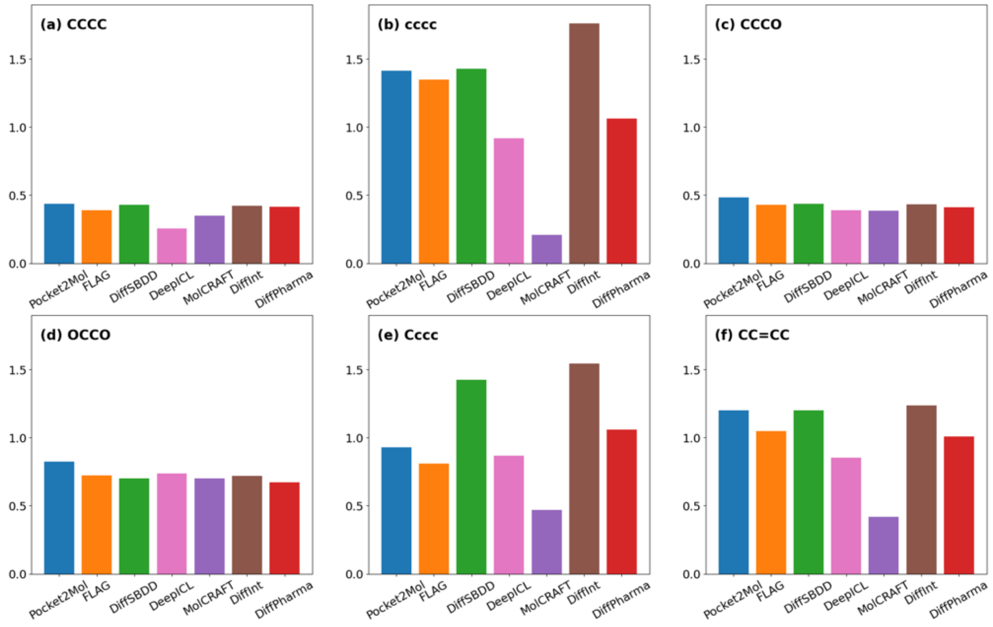

Supplementary Fig. 6: KL divergences between the dihedral angle distributions of the test set and the molecules generated for each model. Figures generated using Matplotlib.

Table 8: KL divergences values of bond lengths, bond angle distributions, and dihedral angles.

		Pocket2Mol	FLAG	DiffSBDD	DeepICL	MolCRAFT	DiffInt	DiffPharma
Bond length	C-C(sp3)	1.36	1.46	0.87	0.51	0.62	1.03	0.91
	C-C(sp2)	1.42	1.25	1.37	1.55	1.56	1.37	1.27
	C-N	1.17	1.01	0.89	0.83	0.92	1.07	0.98
	C-O	1.17	1.16	1.00	0.96	0.68	1.17	1.01
	C=O	1.15	1.23	0.86	1.26	0.66	0.99	0.86
	C-C(aromatic)	0.63	0.53	0.43	0.88	1.37	0.50	0.38
Bond angle	CCC	0.36	0.48	0.51	0.35	0.27	0.56	0.58
	CCO	0.64	0.81	0.68	0.56	0.14	0.90	0.74
	CNC	0.14	0.23	0.43	0.14	0.38	0.56	0.48
	OPO	0.19	0.50	0.38	0.19	0.11	0.37	0.35
	NCC	0.50	0.46	0.51	0.35	0.15	0.67	0.59
	CC=O	0.40	0.36	0.58	0.21	0.20	0.81	0.66
	COC	0.54	0.45	0.62	0.47	0.19	0.76	0.66
Dihedral angle	CCCC	0.44	0.39	0.43	0.25	0.35	0.42	0.42
	cccc	1.41	1.35	1.43	0.92	0.21	1.76	1.06
	CCCO	0.48	0.43	0.44	0.39	0.39	0.43	0.41
	OCCO	0.82	0.72	0.70	0.74	0.70	0.72	0.67
	Cccc	0.93	0.81	1.42	0.87	0.47	1.55	1.06
	CC=CC	1.20	1.05	1.20	0.85	0.42	1.23	1.01

5.4 Pose and Affinity Evaluation

To evaluate the predicted affinity and pose validity of molecules generated within the target protein pocket, gnina is employed for scoring. Conventional docking procedures often involve conformational resampling, which may cause the generated conformations to deviate significantly from the original pose generated by the model. Consequently, such methods make it difficult to assess the validity of the generated pose themselves. To avoid this issue, only minimal energy relaxation is applied to resolve minor steric clashes between the generated molecule and the protein, avoiding large structural rearrangements. Specifically, 200 iterations of gradient descent minimization are performed with a force tolerance set to 10. All affinity and conformation evaluations in this study are conducted under this minimal relaxation setting.

As shown in Supplementary Table 9 and Fig. 7, DiffPharma achieves high scores on both the CNN affinity (prediction of binding affinity) and CNN pose (pose validity) metrics. These results suggest that the 3D structures generated by DiffPharma within the pocket exhibit relatively favorable binding tendencies while maintaining poses consistent with the geometry of the binding site.

It should be noted that the these scores are not obtained under strictly identical evaluation conditions across all methods. For MolCRAFT, the evaluated structures are poses optimized via docking of the generated 3D molecules. In contrast, other generation models, including DiffPharma, evaluate the poses generated directly by the model itself. Although relaxation processing using gnina is performed under identical conditions for all models, these results should be interpreted as a reference metric rather than a quantitative ranking of performance.

Table 9: gnina-based CNN affinity and CNN pose scores.

	CNN affinity (↑)	CNN pose (↑)
Pocke2Mol	4.83	0.61
FLAG	4.73	0.45
DiffSBDD	5.31	0.47
DeepICL	5.06	0.43
MolCRAFT	5.35	0.66
DiffInt	4.91	0.37
DiffPharma	5.17	0.60

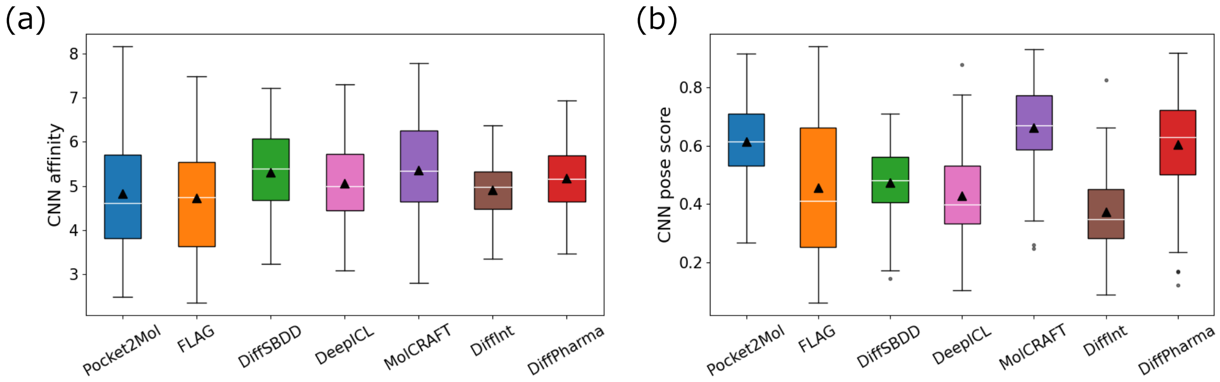

Supplementary Fig. 7: Distributions of (a) CNN affinity and (b) CNN pose scores evaluated using gnina for the generated molecules of each model. Figures generated using Matplotlib.

6 Retrosynthetic pathway

The five generated molecules for each of the drug discovery targets, AKT serine/threonine kinase 1 (AKT1, PDB ID: 3CQW) and serine beta-lactamase (AMPC, PDB ID: 1L2S), which are shown in Fig. 3 of the main text, are subjected to retrosynthetic analysis using SciFinder^n[15]. The resulting retrosynthetic pathways for the molecules targeting 3CQW and 1L2S are presented in Supporting Figs. 8 and 9, respectively.

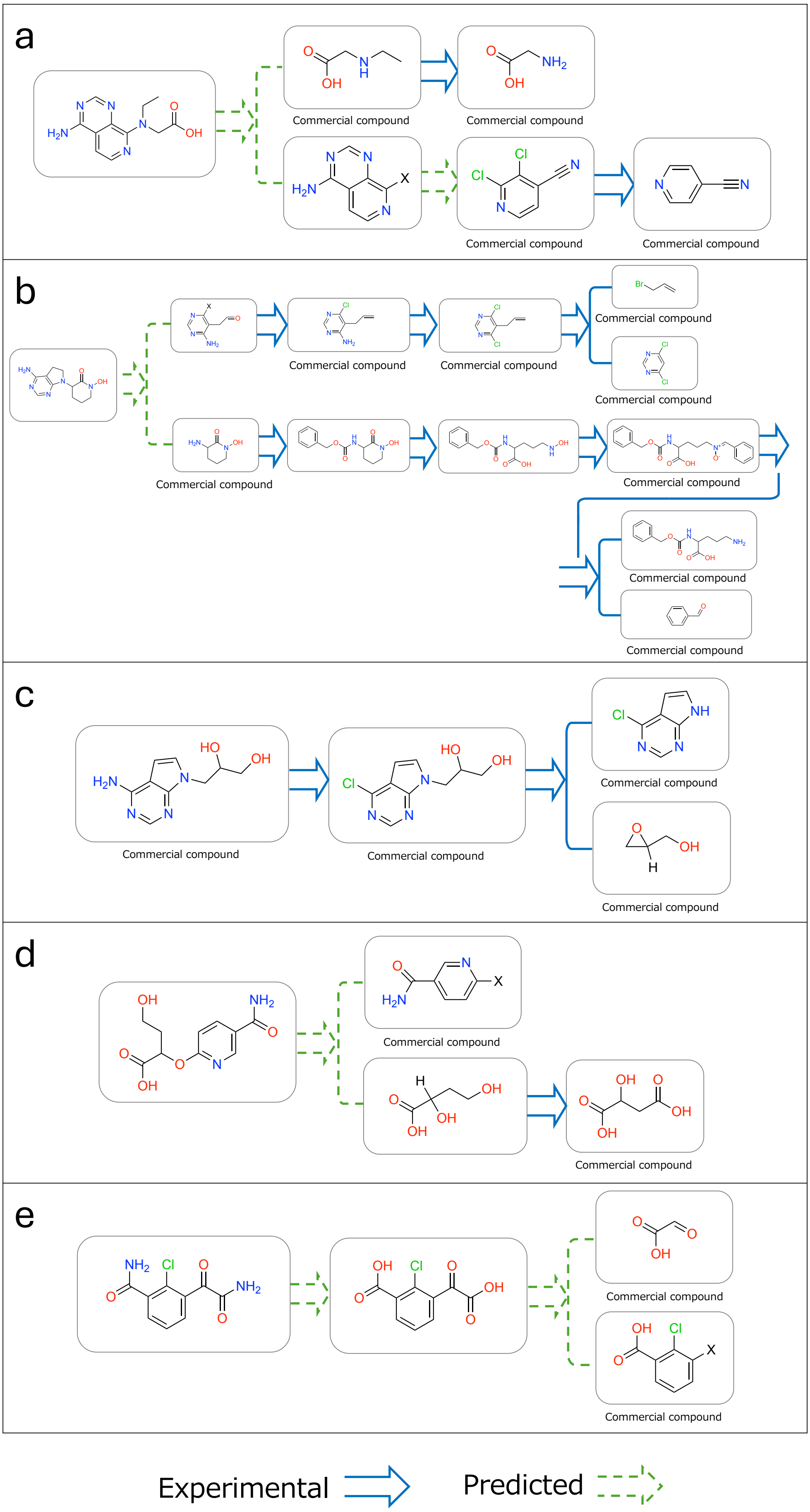

Supplementary Fig. 8: The Predicted retrosynthetic pathway for generated molecules targeting the protein 3CQW. Figures generated using ChemDraw [16].

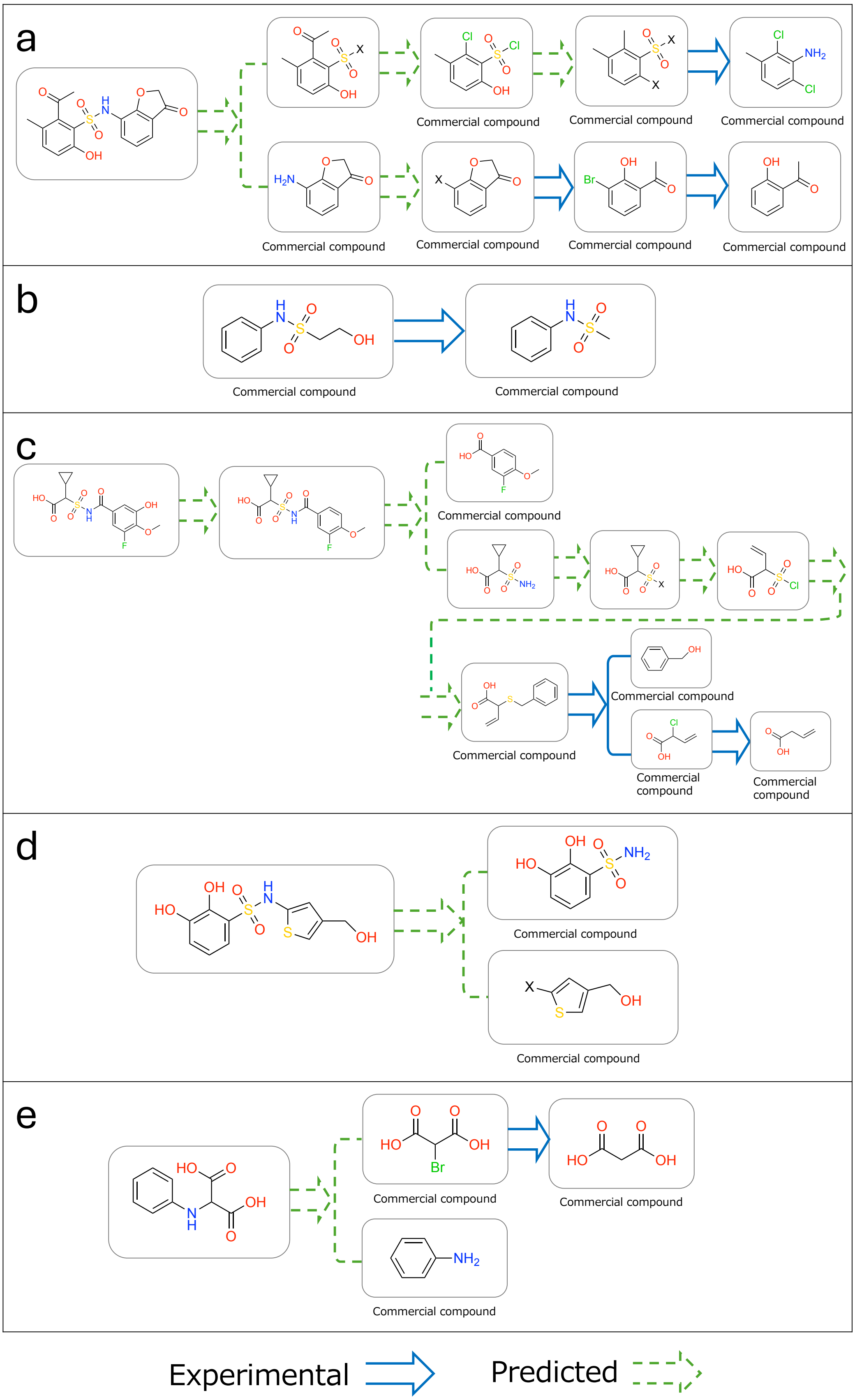

Supplementary Fig. 9: The predicted retrosynthetic pathway for generated molecules targeting the protein 1L2S. Figures generated using ChemDraw.

7 Interaction Reproducibility Analysis for M^{pro}

The training set contains SARS-CoV protease complexes, which share high sequence identity (96%) with SARS-CoV-2 M^{pro}. However, the interaction pattern extracted from the 7GBL SARS-CoV-2 complex and used in this case study does not appear in the training set. Therefore, the M^{pro} case study evaluates the model under a previously unseen interaction pattern.

The molecules generated under the GLU+ and GLU- conditions defined in Section 2.4 are analyzed to quantify how well the specified hydrogen bond and hydrophobic interaction constraints are reproduced. The GLU+ condition applies all seven interaction constraints, whereas GLU- removes only the GLU166 hydrogen-bond constraint. Comparing these two settings enables an assessment of both the reproducibility of individual interaction constraints and the model's robustness to partially incomplete constraint specifications.

In the GLU+ condition, the reproduction rates for the hydrogen-bond interaction constraints are GLU166: 58%, THR190: 45%, ARG188: 35%, and GLN192: 22%, while those for the hydrophobic interaction constraints are MET165: 95%, ASN142: 37% and LEU167: 27%. These values correspond to cosine similarities of 0.50 for hydrogen bonds and 0.60 for hydrophobic interactions. In the GLU- condition, the reproduction rates for the hydrogen-bond interaction constraints are GLU166: 18%, THR190: 46%, ARG188: 38%, and GLN192: 21%, while those for the hydrophobic interaction constraints are are MET165: 97%, ASN142: 35% and LEU167: 21%. The corresponding cosine similarities were 0.40 for hydrogen bonds and 0.59 for hydrophobic interactions. These interaction frequencies for both GLU+ and GLU- are visualized in Supplementary Fig. 10.

Notably, removing the GLU166 hydrogen-bond constraint leads to a pronounced reduction only in the reproduction of the GLU166 interaction itself, whereas the reproduction rates of the other hydrogen-bond and hydrophobic constraints remain largely unchanged. This selectivity indicates that DiffPharma responds locally and specifically to the constraint change without disturbing the rest of the interaction pattern.

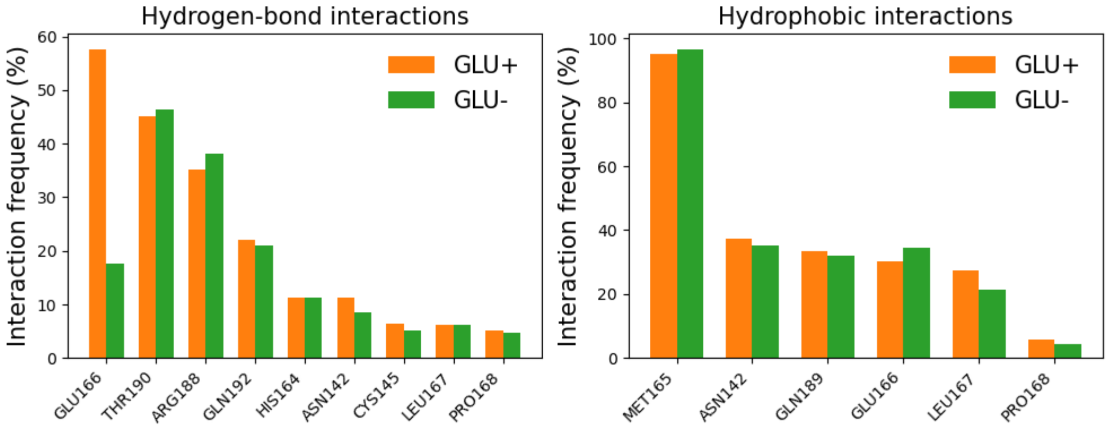

Supplementary Fig. 10: Residue-level interaction reproducibility under GLU+ and GLU- conditions (a) Hydrogen bonds, (b) Hydrophobic interactions. Figures generated using Matplotlib.

8 MD and MM/GBSA settings

8.1 Protein preparation

After removing the ligand molecule and water molecules from the crystal structure, then the protein is imported into Maestro (Schrödinger Suite 2024-1, Schrödinger, LLC)[17]. The structure is preprocessed using the Protein Preparation Wizard, which involves the addition of hydrogen atoms, the assignment of bond orders, and the adjustment of formal charges. The protonation states of the ionizable residues are determined using Epik at a pH of 7.4±2.0, and the missing side chains are modeled using the Prime module. The generated ligand molecule is merged with the processed protein to construct protein-ligand complex data. The complex data are then placed in a rectangular box with a 10 Å buffer distance using the System Builder tool and solvated with the SPC water model. The system is neutralized with Na^+ ions, and all subsequent simulations performed using the OPLS_2005 force field.

8.2 MD simulation

Molecular dybamics (MD) simulations are performed using the Desmond module in Maestro (Schrödinger Suite 2024-1, D. E. Shaw Research)[18]. Long-range electrostatic interactions are computed using the particle-mesh Ewald (PME) method, whereas short-range van der Waals and Coulombic interactions are treated with a cutoff of 9.0 Å.

The solvated system constructed using the System Builder is optimized with the following five relaxation protocols. The first two stages are carried out under an NVT ensemble, consisting of 100 ps of Brownian dynamics at 10 K, followed by 12 ps of Langevin dynamics at 10 K. The final three stages are conducted in an NPT ensemble, with 12 ps at 10 K, 12 ps at 300 K, and then 24 ps without any constraints.

Following the relaxation phase, a production run of 20 ns is implemented under the NPT ensemble at 300 K and 1.01325 bar. Periodic boundary conditions are applied, and the temperature and pressure are controlled using a Nosé–Hoover chain thermostat (with a relaxation time of 1.0 ps) and a Martyna–Tobias–Klein barostat (with a relaxation time of 2.0 ps and isotropic coupling), respectively. Time integration is performed using a multitime-step method, with 2.0 fs for coupled and short-range noncoupled interactions and 6.0 fs for long-range noncoupled interactions. Snapshots of the trajectory are recorded every 20 ps, resulting in a total of 1000 frames.

8.3 MM/GBSA analysis

The binding free energy is estimated using molecular mechanics generalized born surface area (MM/GBSA) calculations implemented in the Prime module (Schrödinger Suite 2024-1, Schrödinger, LLC)[19]. From the 20 ns MD trajectory, the final 2 ns (corresponding to frames 900 to 1000) are extracted and used as representative conformations sampled from the latter phase of the simulation. Each frame is subjected to local energy minimization, and the MM/GBSA binding free energy is computed using the thermal_mmgbsa.py script provided in the Schrödinger Suite.

9 MD simulation results for the generated molecules

The MD simulation results are summarized for the molecules generated under the GLU+ and GLU- conditions, which are defined in the main text (Section 2.4). Supplementary Figs. 11 and 12 show the trajectory analyses for the top 10 molecules generated under the GLU+ condition, ranked by ΔG_{bind}, whereas Supplementary Figs. 13 and 14 present the corresponding results for the GLU- condition. The trajectory analyses include the root-mean-square deviations (RMSDs) of the ligand; the key protein–ligand interaction occupancies throughout the MD simulation; and molecular properties such as the MM-GBSA binding free energy (ΔG_{bind}), QED, and similarity to the reference ligand (smi).

The average interaction occupancies observed during the MD simulations are shown in Supplementary Fig. 15. Notable differences are observed between the GLU+ and GLU- conditions, particularly for interactions with the GLU166 and GLN192 residues. The hydrogen bond with GLU166 is maintained with an occupancy rate of 60.1% under the GLU+ condition, whereas it is 23.3% under the GLU- condition. Similarly, the occupancies for the hydrogen bond with GLN192 are 75.7% for GLU+ and 43.5% for GLU-.

With respect to the π–π stacking interactions with HIS41 and HIS164, the occupancy for HIS41 exceededs that of the reference molecule, whereas HIS164 exhibits lower occupancy. Although the π–π stacking interactions are not explicitly incorporated into the constraints, the preservation of the aromatic ring under the hydrophobic interaction constraint indirectly contributes to the reproduction of the π–π stacking interaction.

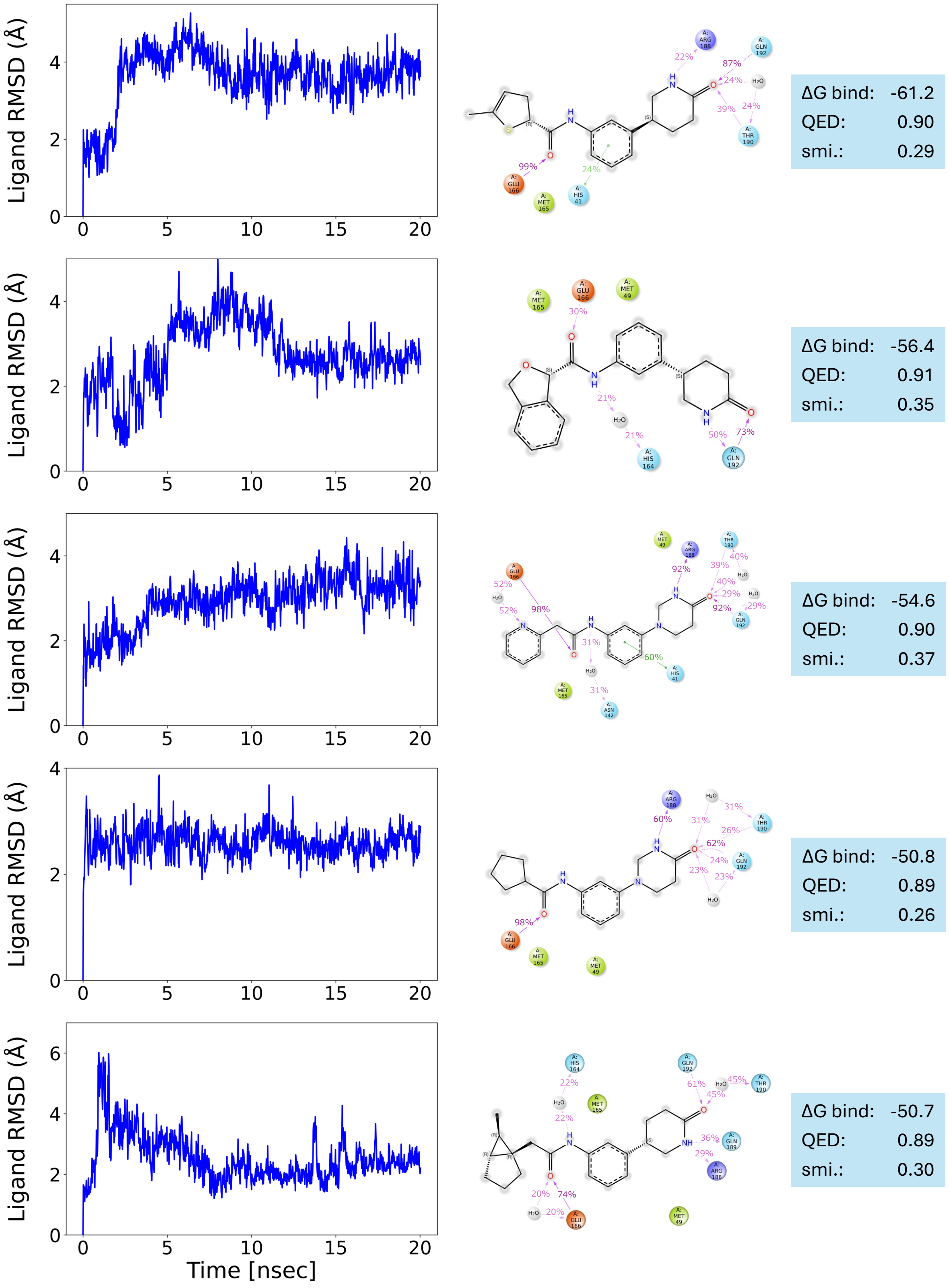

Supplementary Fig. 11: MD simulation results for the top 5 ligands generated under the GLU+ condition ranked by their ΔG_{bind} values. Each panel shows the ligand RMSD plot, the key protein-ligand interactions with occupancy rates exceeding 30% and molecular properties (ΔG_{bind}, QED, and similarity to the reference ligand). Figures generated using Matplotlib and Maestro[20].

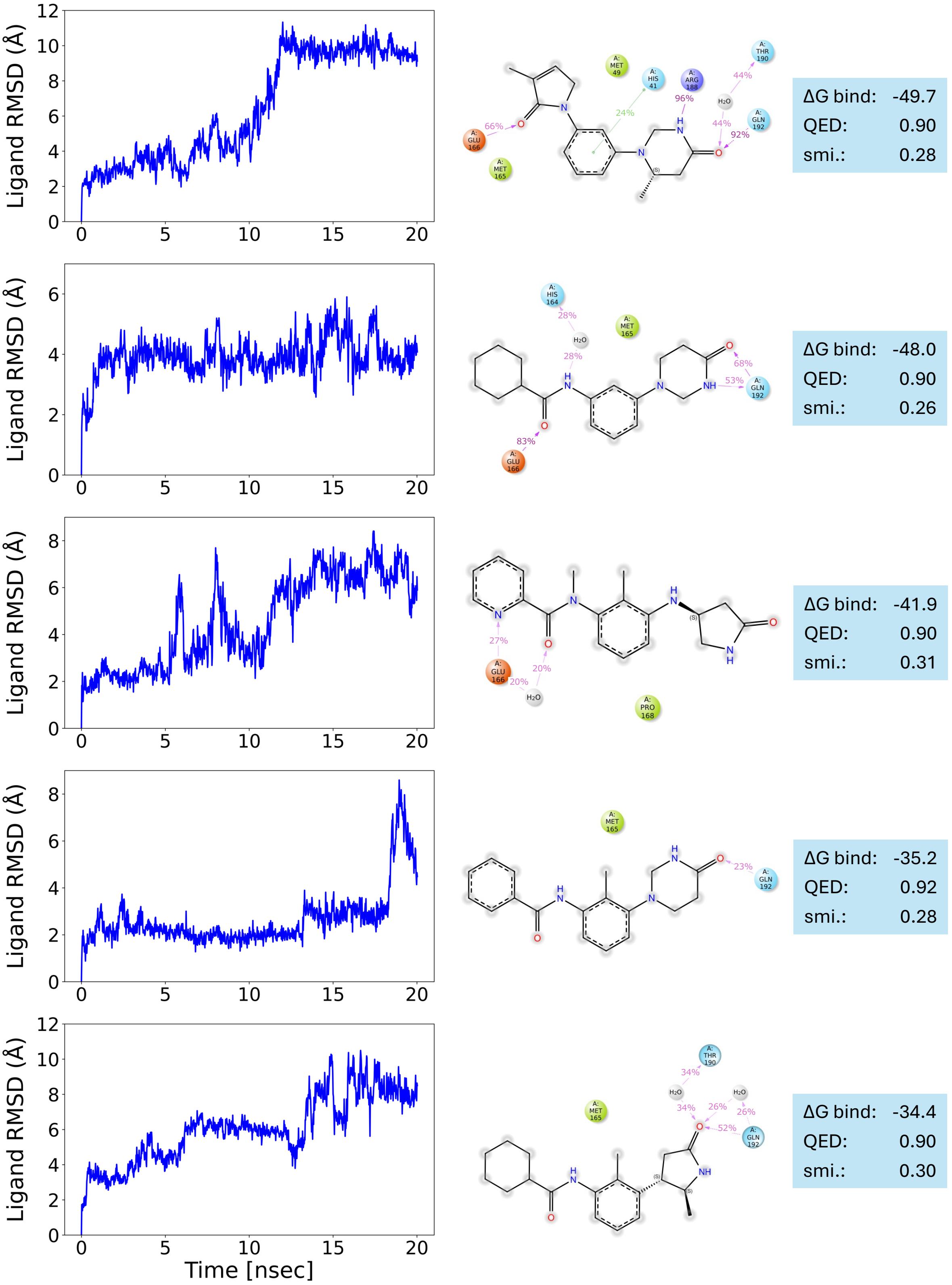

Supplementary Fig. 12: MD simulation results for ligands generated under the GLU+ condition ranked from 6th to 10th based on their ΔG_{bind} values. Each panel shows the ligand RMSD plot, the key protein-ligand interactions with occupancy rates exceeding 30% and molecular properties (ΔG_{bind}, QED, and similarity to the reference ligand). Figures generated using Matplotlib and Maestro.

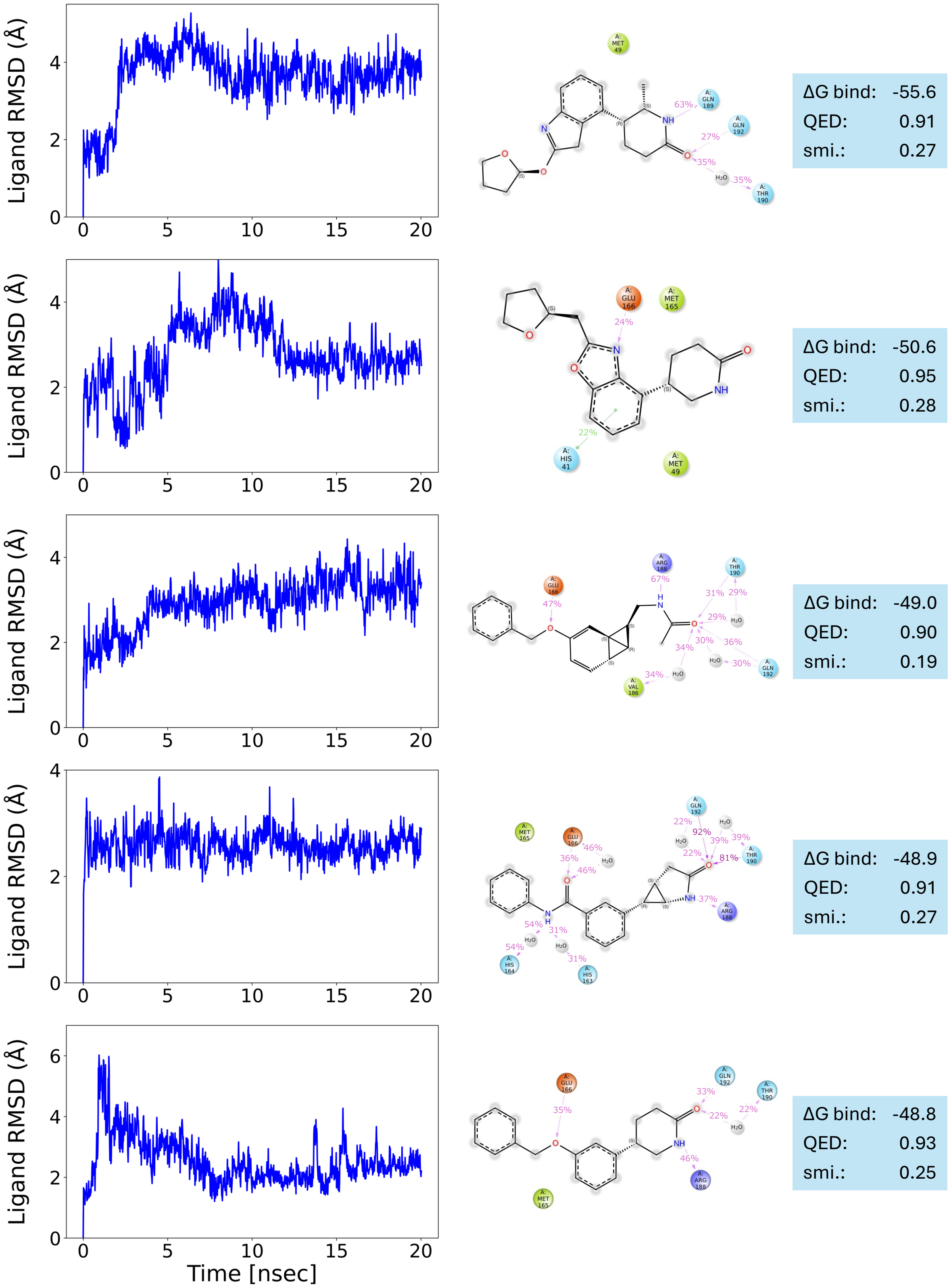

Supplementary Fig. 13: MD simulation results for the top 5 ligands generated under the GLU- condition ranked by their ΔG_{bind} values. Each panel shows the ligand RMSD plot, the key protein-ligand interactions with occupancy rates exceeding 30% and molecular properties (ΔG_{bind}, QED, and similarity to the reference ligand). Figures generated using Matplotlib and Maestro.

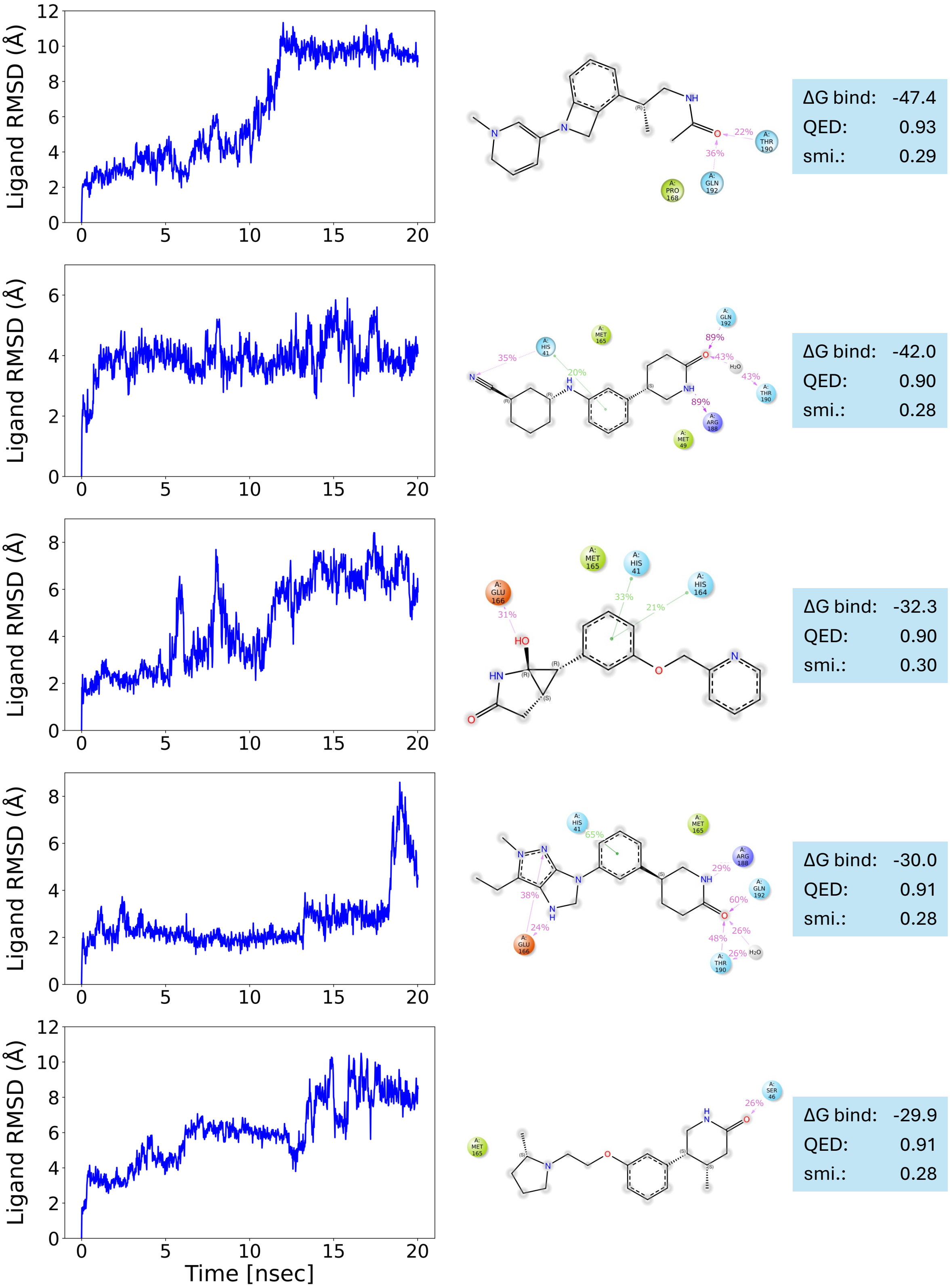

Supplementary Fig. 14: MD simulation results for ligands generated under the GLU- condition ranked from 6th to 10th based on their ΔG_{bind} values. Each panel shows the ligand RMSD plot, the key protein-ligand interactions with occupancy rates exceeding 30% and molecular properties (ΔG_{bind}, QED, and similarity to the reference ligand). Figures generated using Matplotlib and Maestro.

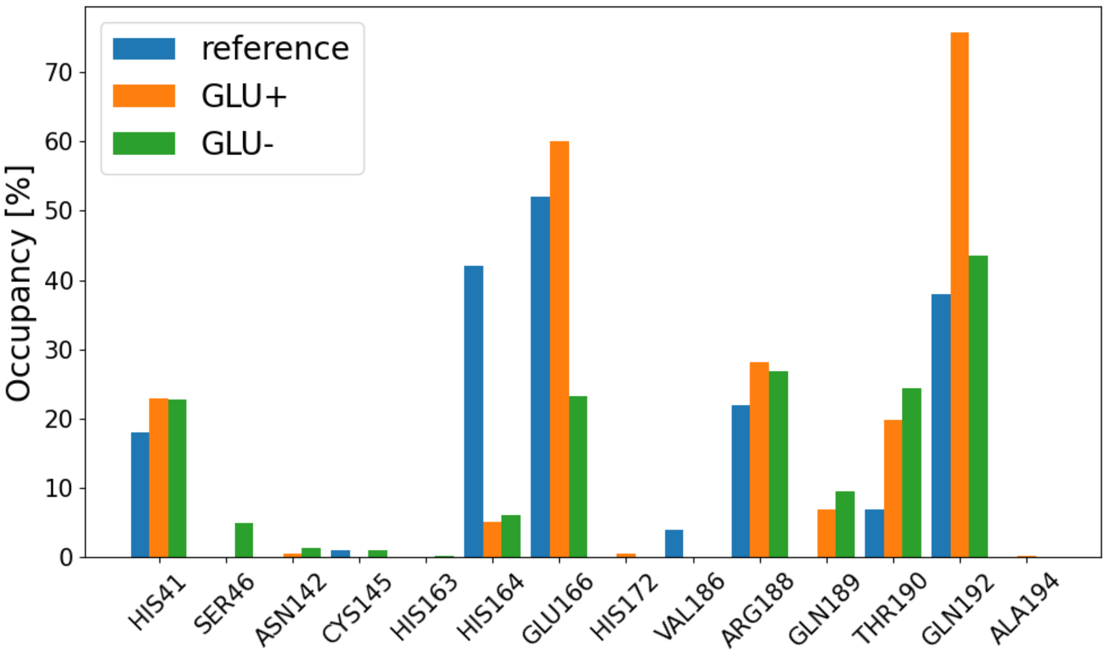

Supplementary Fig. 15: Key protein-ligand interaction occupancy rates for the reference molecules and the molecules generated under GLU+ and GLU- conditions. The results produced for the generated molecules are the averages of 10 molecules. Figures generated using Matplotlib.

References

[1] Schneuing, A., Harris, C., Du, Y., Didi, K., Jamasb, A., Igashov, I., Du, W., Gomes, C., Blundell, T.L., Lio, P., *et al.*: Structure-based drug design with equivariant diffusion models. Nature Computational Science **4**(12), 899–909 (2024)

[2] Wójcikowski, M., Zielenkiewicz, P., Siedlecki, P.: Open drug discovery toolkit (oddt): a new open-source player in the drug discovery field. Journal of cheminformatics **7**, 1–6 (2015)

[3] Hunter, J.D.: Matplotlib: A 2d graphics environment. Computing in Science & Engineering **9**(3), 90–95 (2007) https://doi.org/10.1109/MCSE.2007.55

[4] Qu, Y., Qiu, K., Song, Y., Gong, J., Han, J., Zheng, M., Zhou, H., Ma, W.-Y.: Molcraft: structure-based drug design in continuous parameter space. arXiv preprint arXiv:2404.12141 (2024)

[5] Sako, M., Yasuo, N., Sekijima, M.: Diffint: A diffusion model for structure-based drug design with explicit hydrogen bond interaction guidance. Journal of Chemical Information and Modeling **65**(1), 71–82 (2024)

[6] Schneuing, A., Du, Y., Harris, C., Jamasb, A., Igashov, I., Du, W., Blundell, T., Lió, P., Gomes, C., Welling, M., et al.: Structure-based drug design with equivariant diffusion models. arXiv preprint arXiv:2210.13695 (2022)

[7] Peng, X., Luo, S., Guan, J., Xie, Q., Peng, J., Ma, J.: Pocket2mol: Efficient molecular sampling based on 3d protein pockets. In: International Conference on Machine Learning, pp. 17644–17655 (2022). PMLR

[8] ZHANG, Z., Min, Y., Zheng, S., Liu, Q.: Molecule generation for target protein binding with structural motifs. In: The Eleventh International Conference on Learning Representations (2023). https://openreview.net/forum?id=Rq13idF0F73

[9] Zhung, W., Kim, H., Kim, W.Y.: 3d molecular generative framework for interaction-guided drug design. Nature Communications **15**(1), 2688 (2024)

[10] Bickerton, G.R., Paolini, G.V., Besnard, J., Muresan, S., Hopkins, A.L.: Quantifying the chemical beauty of drugs. Nature chemistry **4**(2), 90–98 (2012)

[11] Wildman, S.A., Crippen, G.M.: Prediction of physicochemical parameters by atomic contributions. Journal of chemical information and computer sciences **39**(5), 868–873 (1999)

[12] Lipinski, C.A., Lombardo, F., Dominy, B.W., Feeney, P.J.: Experimental and computational approaches to estimate solubility and permeability in drug discovery and development settings. Advanced drug delivery reviews **23**(1-3), 3–25 (1997)

[13] Ertl, P., Schuffenhauer, A.: Estimation of synthetic accessibility score of drug-like molecules based on molecular complexity and fragment contributions. Journal of cheminformatics **1**, 1–11 (2009)

[14] RDKit: Open-source cheminformatics (2006). http://www.rdkit.org

[15] Gabrielson, S.W.: Scifinder. Journal of the Medical Library Association: JMLA **106**(4), 588 (2018)

[16] Revvity Signals Software, I.: ChemDraw Professional. Software for chemical structure drawing and analysis. www.revvity.com

[17] Madhavi Sastry, G., Adzhigirey, M., Day, T., Annabhimoju, R., Sherman, W.: Protein and ligand preparation: parameters, protocols, and influence on virtual screening enrichments. Journal of computer-aided molecular design **27**, 221–234 (2013)

[18] Bowers, K.J., Chow, E., Xu, H., Dror, R.O., Eastwood, M.P., Gregersen, B.A., Klepeis, J.L., Kolossvary, I., Moraes, M.A., Sacerdoti, F.D., *et al.*: Scalable algorithms for molecular dynamics simulations on commodity clusters. In: Proceedings of the 2006 ACM/IEEE Conference on Supercomputing, p. 84 (2006)

[19] Jacobson, M.P., Pincus, D.L., Rapp, C.S., Day, T.J., Honig, B., Shaw, D.E., Friesner, R.A.: A hierarchical approach to all-atom protein loop prediction. Proteins: Structure, Function, and Bioinformatics **55**(2), 351–367 (2004)

[20] Schrödinger, LLC: Schrödinger Release 2025-4: Maestro, New York, NY (2025)
